# Challenging the *Wigglesworthia*, *Sodalis*, *Wolbachia* symbiosis dogma in tsetse flies: *Spiroplasma* is present in both laboratory and natural populations

**DOI:** 10.1038/s41598-017-04740-3

**Published:** 2017-07-05

**Authors:** V. Doudoumis, F. Blow, A. Saridaki, A. Augustinos, N. A. Dyer, I. Goodhead, P. Solano, J.-B. Rayaisse, P. Takac, S. Mekonnen, A. G. Parker, A. M. M. Abd-Alla, A. Darby, K. Bourtzis, G. Tsiamis

**Affiliations:** 1 Department of Environmental and Natural Resources Management, University of Patras, Agrinio, 30100 Greece; 2 Department of Functional and Comparative Genomics, Institute of Integrative Biology, University of Liverpool, Crown Street, Liverpool, L69 7ZB UK; 3 Insect Pest Control Laboratory, Joint FAO/IAEA Division of Nuclear Techniques in Food and Agriculture, Vienna International Centre, P.O. Box 100, 1400 Vienna, Austria; 4 Liverpool School of Tropical Medicine, Pembroke Place, Liverpool, L3 5QA UK; 5 Institut de Recherche pour le Développement, Unité Mixte de Recherche Interactions Hôtes-Vecteurs-Parasites-Environnement dans les Maladies Tropicales Négligées Dues aux Trypanosomatides, 34398 Montpellier, France; 6 Centre International de Recherche-Développement sur l’Élevage en zone Subhumide (CIRDES), N°559, Rue 5-31 Avenue du Gouverneur Louveau, 01 BP 454 Bobo Dioulasso 01, Burkina Faso; 7 Institute of Zoology, Section of Molecular and Applied Zoology, Slovak Academy of Sciences, Dubravska cesta 9, 845 06 Bratislava, Slovakia; 8 National Institute for Control and Eradication of Tsetse and Trypanosomosis (NICETT), Kality Centre, Akaki Kality, Addis Ababa, Ethiopia; 9 Scientica, Ltd, Hybesova 33, 831 06 Bratislava, Slovakia; 10 School of Environment and Life Sciences, The Crescent, University of Salford, Salford, M5 4WT UK

## Abstract

Profiling of wild and laboratory tsetse populations using 16S *rRNA* gene amplicon sequencing allowed us to examine whether the “*Wigglesworthia*-*Sodalis*-*Wolbachia* dogma” operates across species and populations. The most abundant taxa, in wild and laboratory populations, were *Wigglesworthia* (the primary endosymbiont), *Sodalis* and *Wolbachia* as previously characterized. The species richness of the microbiota was greater in wild than laboratory populations. *Spiroplasma* was identified as a new symbiont exclusively in *Glossina fuscipes fuscipes* and *G*. *tachinoides*, members of the *palpalis* sub-group, and the infection prevalence in several laboratory and natural populations was surveyed. Multi locus sequencing typing (MLST) analysis identified two strains of tsetse-associated *Spiroplasma*, present in *G*. *f*. *fuscipes* and *G*. *tachinoides*. *Spiroplasma* density in *G*. *f*. *fuscipes* larva guts was significantly higher than in guts from teneral and 15-day old male and female adults. In gonads of teneral and 15-day old insects, *Spiroplasma* density was higher in testes than ovaries, and was significantly higher density in live versus prematurely deceased females indicating a potentially mutualistic association. Higher *Spiroplasma* density in testes than in ovaries was also detected by fluorescent *in situ* hybridization in *G*. *f*. *fuscipes*.

## Introduction

Tsetse (*Glossina* spp.; Diptera: Glossinidae) are viviparous, obligate blood feeding flies found in sub-Saharan Africa. They are the only cyclical vectors of African trypanosomes, responsible for human African trypanosomosis (HAT) and animal African trypanosomosis (AAT)^1, 2^. Tsetse larvae feed on milk produced in the milk glands of their mothers, pupariating less than an hour after birth. Adult flies of both sexes feed exclusively on largely sterile blood meals.

The microbiota of tsetse flies is of interest because of their unique lifestyle, highlighted by their bilateral transmission, and reproductive strategy, including the elicitation of phenotypes like cytoplasmic incompatibility, as well as its potential for vector and disease control^3–5^. So far, it is known that tsetse flies harbour three main symbiotic microbes: *Wigglesworthia*, *Sodalis* and *Wolbachia*. These three symbionts form the tsetse symbiosis dogma. The primary mutualist symbiont *Wigglesworthia* provides dietary supplements that are necessary for host fecundity as well as supporting larval development and the maturation process of the adult immune system^6–9^. The facultative symbiont *Sodalis* is present in tsetse populations with a putative role in the ability to transmit trypanosomes^10^. Finally, *Wolbachia* has been found in natural populations of tsetse flies with some species exhibiting up to 100% infection rate^11, 12^, while others have been found to be free of *Wolbachia*, like *G*. *p*. *palpalis* (*Gpp*)^12^. In addition, the *Wolbachia* strain present in *Glossina morsitans morsitans* (*Gmm*) can induce cytoplasmic incompatibility under laboratory conditions^11^.

There have been a limited number of culture-dependent and culture-independent studies aiming to characterize the microbiota associated with tsetse flies. Using classical microbiological approaches, Geiger and colleagues isolated *Acinetobacter*, *Enterobacter*, *Enterococcus*, *Providencia*, *Sphingobacterium*, *Chryseobacterium*, *Lactococcus*, *Staphylococcus* and *Pseudomonas* species from the guts of field collected *Gpp* in Cameroon^13–16^. They also isolated a new bacterial species, *Serratia glossinae*, from the midgut of *G*. *palpalis gambiensis* (*Gpg*) collected in Burkina Faso^14^. A screen for both cultivable and non-cultivable bacteria in whole *G*. *fuscipes fuscipes* (*Gff*) was performed with flies collected in Kenya^17^. *Firmicutes*, and particularly members of the *Bacillus* genus, were identified as the most dominant group while *Paenibacillus*, *Staphylococcus* and *Exiguobacterium* spp. were also isolated at lower density. *Gammaproteobacteria* were also present, mainly members of the *Enterobacteriaceae* family like *Morganella* and *Providencia* and to a lesser degree *Pseudomonas* spp., while *Burkholderia* was the only member of *Betaproteobacteria* detected in this study^17^. Using a culture independent approach, beyond the mutualist symbiont *Wigglesworthia*, only *Bacillus* and *Serratia* spp. were additionally detected^17^. Aksoy and colleagues sampled guts of Ugandan *Gff*, *Gmm*, and *G*. *pallidipes* (*Gpal*) tsetse flies, and profiled the microbiota using Illumina amplicon sequencing^18^. *Wigglesworthia* was the dominant taxon, while *Sodalis* was generally detected at low density (<0.05%). However, a small number of flies harboured high levels of *Sodalis* and *Serratia* spp. Νon-*Wigglesworthia Enterobacteriaceae* together with *Halomonas* spp. were also found at lower abundance at all field sites studied, with some bacterial taxa being unique to a sample site.

*Spiroplasma* is a genus of wall-less bacteria belonging to the class *Mollicutes* and it has been associated with diverse plants and arthropods^19–22^. *Spiroplasma* is grouped into three major clades as has been shown by 16S *rRNA* gene-based as well as multi locus sequence typing (MLST) studies^23–30^. *Spiroplasma* exhibits a dual life, with capacity to live intracellularly in a variety of tissues and systemically in the haemolymph^31^. *Spiroplasma* has developed a wide range of symbiotic associations, producing diverse effects on insect evolution, ecology, reproduction and sex determination. *Spiroplasma* has been found to confer protection against a nematode in *Drosophila neotestacea* ^32^, against fungi in the pea aphid (*Acyrthosiphon pisum*)^33^, and against a parasitoid wasp in *Drosophila hydei* ^34^. *Spiroplasma* can also be pathogenic in plants^35^, insects^36–38^ and crustaceans^39–44^. Moreover, several species of *Spiroplasma* have been associated with reproductive alterations such as male killing^29, 45–48^. Except *Spiroplasma*, other reproductive parasites that have been associated with insects are *Arsenophonus*, *Cardinium*, and *Rickettsia*. *Arsenophonus* is known to establish diverse symbiotic interactions with around 5% of insect species, with the most profound phenotype induced being the son-killer trait^49, 50^. *Cardinium* has been found exclusively to Hymenoptera, Hemiptera, Diptera, and Acari and it is known to induce cytoplasmic incompatibility and feminization^51, 52^. Finally, *Rickettsia* has been associated with regulating insect growth and immunity to pathogenic fungi^53–55^.

In this study we employed high throughput sequencing of the 16S *rRNA* gene to unravel the diversity of tsetse associated bacteria in a wider variety of species, field and laboratory populations than any previous tsetse microbiota study. We asked whether the “*Wigglesworthia-Sodalis-Wolbachia* dogma” applies across species and populations, and whether the microbiota varies between laboratory and field individuals of the same tsetse species. *Spiroplasma* was identified as a novel symbiont of *Gff* and *G*. *tachinoides* (*Gt*), and infection prevalence was surveyed in laboratory and natural populations. Quantitative PCR was used to characterize its density in different developmental stages and tissues, and to quantify infection levels in collapsing mass-rearing tsetse fly colonies. Fluorescent *in situ* hybridization (FISH) was used to localize the newly identified symbiont in tissues including the gonads.

## Results

### 16S *rRNA* gene amplicon sequencing reveals novel interspecific diversity in natural populations of tsetse flies

Microbial community composition and diversity of thirty-two whole insects from *G*. *medicorum* (*Gmed*), *G*. *morsitans submorsitans* (*Gms*), *G*. *p*. *gambiensis* (*Gpg*), and *G*. *tachinoides* (*Gt*) collected in Folonzo, Burkina Faso were investigated by 16S *rRNA* gene amplicon sequencing, producing 5,761,899 reads after quality filtering. These reads were combined with a total of 8,300,515 quality-filtered reads generated from 124 whole guts of *Gff*, *Gmm*, *Gpal* from a previous study^18^, which used an identical technical approach for amplicon generation and sequencing. Including the data from the above mentioned study^18^ provided additional *Wigglesworthia*/co-divergence context to our dataset due to the increased host diversity. This approach enabled us to characterize low-frequency, high-abundance taxa. Whole insect samples from *Gpg*, and *Gt* were the most bacterial species-rich samples containing higher numbers of unique OTUs (Supplementary Table 1).

**Figure 1:**
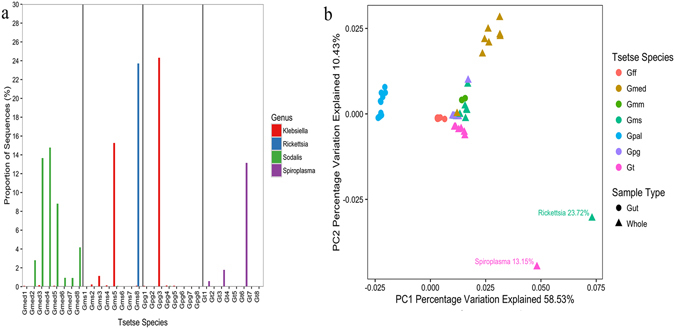
(**a**) Relative abundance of *Klebsiella*, *Rickettsia*, *Sodalis*, and *Spiroplasma* in whole wild tsetse flies. (Gmed: *G*. *medicorum*; Gms: *G*. *morsitans submorsitans*; Gpg: *G*. *p*. *gambiensis*; Gt: *G*. *tachinoides*). (**b**) Weighted Unifrac Principal Component Analysis of 16 S *rRNA* gene MiSeq data. Each data point represents an individual tsetse fly and is coloured according to *Glossina* species. Convergent evolution between the primary endosymbiont *Wigglesworthia* and its host due to direct vertical transmission generates a tsetse species-clustering pattern that simplifies the detection of emergent taxa such as *Spiroplasma* and *Rickettsia*. All gut samples originated from the study by Aksoy *et al*. in 2014, and whole samples were collected in Burkina Faso. (Gmm: *Glossina morsitans morsitans*; Gff: *G*. *fuscipes fuscipes*; Gmed: *G*. *medicorum*; Gms: *G*. *morsitans submorsitans*; Gpal: *G*. *pallidipes*; Gpg: *G*. *palpalis gambiensis*; Gt: *G*. *tachinoides*).

The primary nutritional endosymbiont of tsetse flies *Wigglesworthia glossinidia* was the most abundant taxon in all samples, and constituted between 71 and 99% of the total community in each individual. Variation in the relative abundance of *W*. *glossinidia* was due to the heterogeneous distribution of secondary taxa, which varied in infection frequency and abundance between individuals in both an intra- and inter-specific fashion (Fig. 1a). Secondary taxa included the facultative symbionts *S*. *glossinidius* and *Wolbachia*, alongside *Spiroplasma*, which have not previously been reported in tsetse flies. The relative abundance of secondary taxa was highly variable (from <0.01% to 28%) depending upon the genus of the bacterium and the species of *Glossina* (Fig. 1a). This contributed to the variation in bacterial community composition between *Glossina* species. Clustering by species is illustrated in Fig. 1b, where Principal Component 1 and Principal Component 2 describe 58.53% and 10.84% of the variance respectively. Clustering can be partly attributed to the co-diversification of *Wigglesworthia*, which is the main component of the community, with its tsetse host^56^. For this reason, outliers are conspicuous, as is observed with the two individuals infected with *Spiroplasma* and *Rickettsia* at 13.15% and 23.72% relative abundance respectively (Fig. 1b).

**Figure 2:**
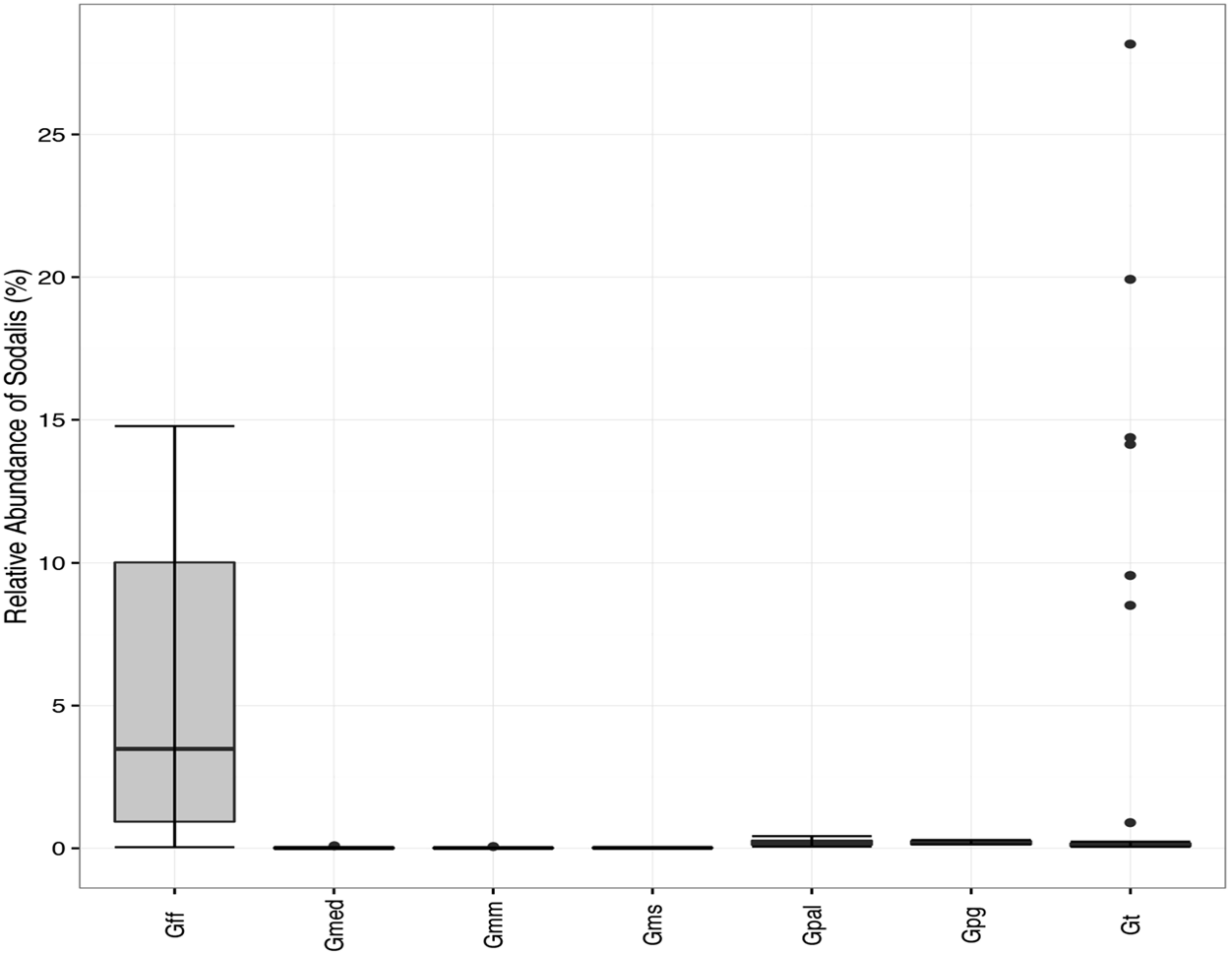
*Sodalis* relative abundance in each tsetse species. Boxes denote the interquartile range, the line within the box is the median, and whiskers extend to the most extreme value within 1.5 *interquartile range. Outliers are indicated as circles. Gff: *G*. *fuscipes fuscipes* (n = 76); Gmed: *G*. *medicorum* (n = 8); Gmm: *G*. *morsitans morsitans* (n = 6); Gms: *G*. *morsitans submorsitans* (n = 8); Gpal: *G*. *pallidipes* (n = 42); Gpg: *G*. *p*. *gambiensis* (n = 8); Gt*: G*. *tachinoides* (n = 8).

*Sodalis* was found at higher frequency and relative abundance in whole *Gmed* and *Gpal* guts (Figs 2 and Supplementary Figure 1). In the other *Glossina* species it has been detected but to a much lower abundance with a relative abundance of 0.5% or less, with *Gms* exhibiting the lowest abundance. *Wolbachia* infections were found infrequently and with low relative abundance of up to 0.04% in any wild sample, with *Gmed* exhibiting the highest infection prevalence (Supplementary Figure 1).

In addition, several other taxa previously associated with tsetse flies were detected including multiple members of the *Enterobacteriaceae*, such as *Klebsiella*, *Erwinia*, *Trabulsiella*, *Pantoea*, and *Serratia*. These infections occurred at low relative abundance, excluding those with *Klebsiella*, which was found to be dominant in one *Gpg* and one *Gms* whole fly at a relative abundance of 24.3% and 15.3% respectively (Fig. 1a). Amplicon profiling was also able to detect taxa that had not previously been associated with the tsetse fly. Several wild individuals of *Gff* and *Gt*, which belong to the *palpalis* subgroup of the *Glossina* genus, were infected with *Spiroplasma*. Relative abundances were generally low (<1%) (Supplementary Table 2), but were found to be as high as 13.2% in one *Gt* whole fly from Burkina Faso (Fig. 1a).

### 16S *rRNA* gene amplicon sequencing of laboratory reared tsetse flies

*Gff*, *Gmm*, and *Gpal* tissue samples from three developmental stages were sequenced, producing 2,445,369 reads after quality filtering. Similarly to wild populations, the three known taxa (*Wigglesworthia*, *Sodalis* and *Wolbachia*) were found in the laboratory flies. However, additional bacterial species were also detected, with members of *Flavobacterium*, *Propiniobacterium*, *Brevundimonas*, *Aeromonas*, and *Rhodospirillales* identified in *Gmm*, *Gff*, and *Gpal*. Sequences related to *Acinetobacter* and *Pantoea* were identified in *Gmm* and *Gpal*. Additionally, sequences related to *Streptococcus* were found in *Gmm*, and *Gff*, while sequences related to *Shewanella*, and *Pedobacter* were discovered only in *Gmm*. Relative abundance was influenced by tissue sample type with gut tissues being enriched for *Wigglesworthia* while reproductive tissues were characterized by the presence of *Wolbachia* and *Sodalis*.

For *Gpal*, the most bacterial species-rich samples were those associated with gonads of teneral flies while gut samples were less species-rich based on both Chao1 and ACE indices (Supplementary Table 3). Gut samples of teneral males and females displayed lower species richness (Supplementary Table 3). The same trend was observed for *Gff*. Gut samples of teneral flies exhibited the lowest species diversity and richness indices, which increased over time (Supplementary Table 3). Conversely, gonads presented a higher diversity and richness index in teneral flies and decreased in aged flies. This pattern was not observed in *Gmm*. Finally, the natural populations exhibited a statistically significant higher species-rich index (Chao1) when compared with the laboratory populations (p < 0.016).

We observed variation in the frequency and relative abundance of *Wolbachia* in lab populations. The mean relative abundance of *Wolbachia* was significantly higher in *Gmm* flies compared with those from the *Gff* or *Gpal* populations (ANOVA, p ≤ 0.01) (Supplementary Table 2). This was due to increased relative abundance of *Wolbachia* in reproductive tissues compared to larval or gut tissues within the *Gmm* population (ANOVA, p ≤ 0.01).

**Figure 3:**
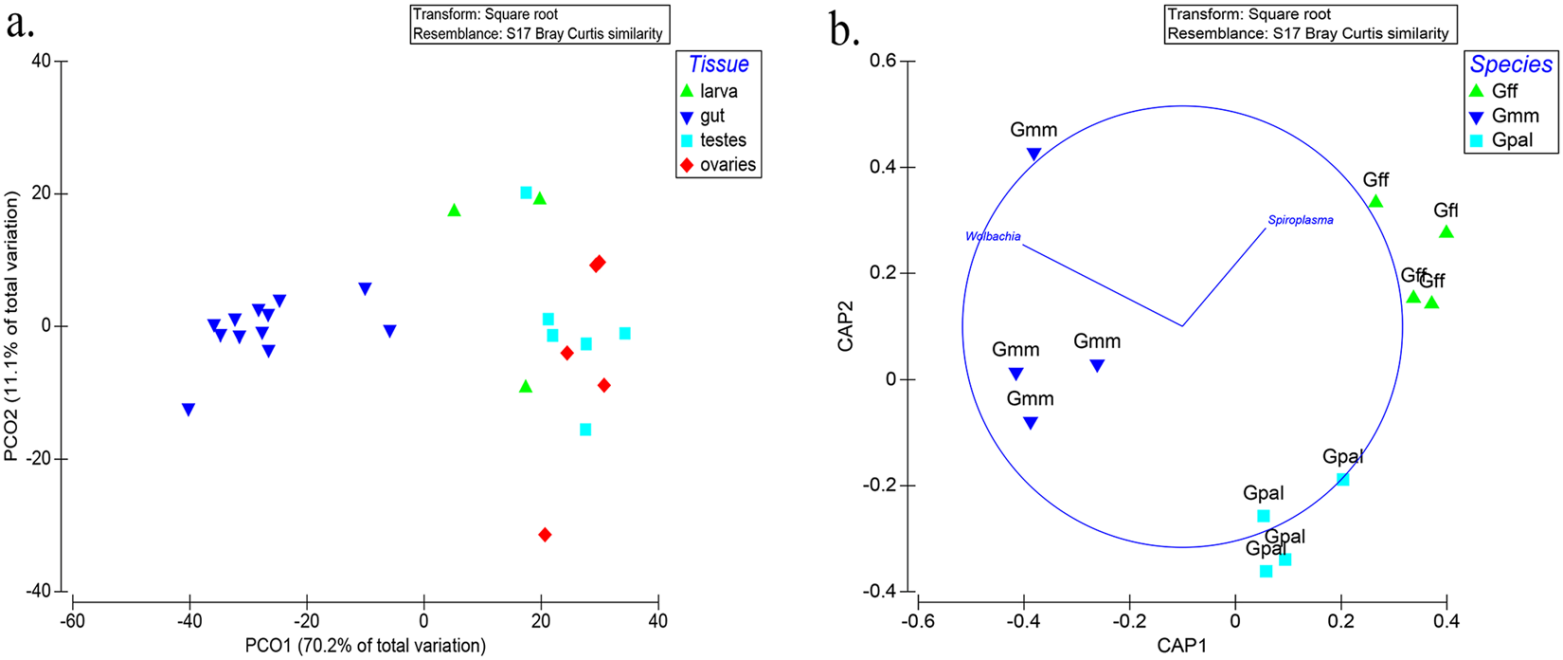
(**a**) Principal coordinate analysis (PCοΑ) of bacterial communities based on relative abundances of OTUs with ordinations from laboratory populations of gut, ovaries, testes and larvae. Variance explained by each PCοΑ axis is given in parentheses. (**b**) Canonical analysis of principal coordinates (CAP) ordinations of gonadal bacterial communities based on relative abundances of OTUs from the laboratory populations. The constrained ordinations show maximized differences among the different *Glossina* species, (Gmm: *Glossina morsitans morsitans*, Gff: *Glossina fuscipes fuscipes*, Gpal: *Glossina pallidipes*). (trace_Q_m’HQ_m_ (0.9598; p < 0.05)).

Bacterial communities were strongly clustered according to the tissue of origin separating the bacterial communities from guts from those from reproductive tissues (Fig. 3a). This factor explained 81.3% of the total variance. Canonical analysis of principal coordinates (CAP), revealed distinct clustering within the gonadal tissue (Fig. 3b). The bacterial communities associated with the gonadal tissue also seem to be statistically affected by the host; *Gmm*, *Gff*, and *Gpal* bacterial communities associated with the reproductive organs clustered separately (Fig. 3b), with *Spiroplasma* driving the *Gff* cluster and *Wolbachia* the *Gmm*. CAP ordinations were supported by significant trace_Q_m’HQ_m_ statistics (0.9598; p < 0.05).

### *Spiroplasma* infection status assessed by PCR screening of natural and laboratory tsetse populations

We used PCR-based screening methods to assay for the presence of four insect reproductive parasites: *Spiroplasma*, *Arsenophonous*, *Rickettsia*, and *Cardinium*, in four *Glossina* species from the laboratory, *Gmm* (n = 19), *Gff* (n = 76), *Gpal* (n = 20), and *Gpg* (n = 19) and wild *Gff* (n = 98). Of the four examined *Glossina* species, *Spiroplasma* infections were found only in *Gff* with an infection ranging from 6.7 to 80% (Table 1), while none of the four tsetse species examined were infected with *Arsenophonus*, *Rickettsia* or *Cardinium*.

**Table 1:** *Spiroplasma* prevalence in ten *Glossina* species.

Species	Origin	Collection Date	Location (Area, Population, Sex)	Tissue	No. of Samples	Spiroplasma Infection Rate (%)
*G*. *austeni*	Field	1995	Tanzania (Zanzibar) 6 Males, 4 NA^a^)	Whole	10	0
	Field	1996	Tanzania (Jozani) Females	Whole	10	0
	Field	1999	South Africa (Zululand) 3 Females, 4 Males, 3 NA	Whole	10	0
	Field	Unknown	Coastal Tanzania (Muhoro) Female	Whole	2	0
*G*. *brevipalpis*	Laboratory	1995	Seibersdorf Laboratory Colony, 8 Females and 8 Males	Whole	16	0
	Laboratory	Unknown	Coastal Tanzania (Pangani), Males	Whole	5	0
*G*. *f*. *fuscipes*	Field	1994	Uganda (Buvuma Island, GFTF2), NA	Whole	17	0
	Field	1994	Uganda (Buvuma Island, GFKF2), NA	Whole	5	0
	Field	1994	Uganda (Buvuma Island, GFFBUV2), NA	Whole	9	0
	Field	1994	Uganda (Buvuma Island, GFFTOR2)^c^, NA	Whole	15	6.7
	Laboratory	1995	Seibersdorf Laboratory Colony^c^, 18 Females, 18 Males	Whole	36	33.4
	Laboratory	2013	Bratislava Laboratory Colony^d^, 20 Females, 20 Males	Whole	40	80
	Field	2014	Uganda (Lukoma-Buvuma Islands, 350)^d^ 20 Females, 32 Males	Whole	52	5.8
*G*. *m*. *centralis*	Laboratory	2008	Yale Laboratory Colony, NA	Whole	1	0
*G*. *m*. *morsitans*	Laboratory	2008	KARI-TRC Laboratory Colony, NA	Whole	15	0
	Laboratory	2010	Antwerp Laboratory Colony, NA	Whole	4	0
*G*. *m*. *submorsitans*	Field	2010	Burkina Laboratory (Folonzo), Females	Whole	8	0
*G*. *pallidipes*	Laboratory	1999	Seibersdorf Laboratory Colony, NA	Whole	2	0
	Laboratory	2008	Seibersdorf Laboratory Colony, 6 Females, 7 Males	Whole	13	0
	Laboratory	Unknown	Uganda-UGA/IAEA, Males	Whole	5	0
*G*. *p*. *gambiensis*	Laboratory	1995	CIRDES Laboratory Colony, 4 Female, 5 Males	Whole	9	0
	Laboratory	2005	CIRDES Laboratory Colony, 1 Females, 9 Males	Whole	10	0
*G*. *p*. *palpalis*	Laboratory	1995	Seibersdorf Laboratory Colony^b^, 8 Females, 8 Males	Whole	16	12.5
*G*. *tachinoides*	Laboratory	1995	Seibersdorf Laboratory Colony^b^, Females	Whole	7	14.3
	Field	2010	Burkina Faso (Folonzo)^d^, Females	Whole	8	37.5

^a^Sex of individuals is not known. ^b^Characterization of *Spiroplasma* infection was based only on 16S *rRNA* gene sequencing. ^c^The Seibersdorf laboratory-colony was established from the Central African Republic in 1986. This colony was transferred to Bratislava, Slovakia in 2009. ^d^Full MLST genotyping.

To examine the distribution of *Spiroplasma*, six additional *Glossina* species were PCR-screened for *Spiroplasma* infection. Only *Gt* and *Gpp* were positive for *Spiroplasma*, and showed an infection rate of 26.7% and 12.5% respectively (Table 1). The PCR screening for *Spiroplasma* infection was further extended to 327 historical and contemporary samples from wild and laboratory colonies representing 10 species of tsetse fly (Table 1). Only members of the *palpalis* subgroup were found infected with *Spiroplasma*, including *Gff*, *Gpp* and *Gt*, with a prevalence ranging from 6% to 80%. Notably, the prevalence was higher in laboratory colonies than natural populations, and some populations demonstrated a disparity in infection between sexes (Table 1).

### Genotyping of *Spiroplasma* strains

**Figure 4:**
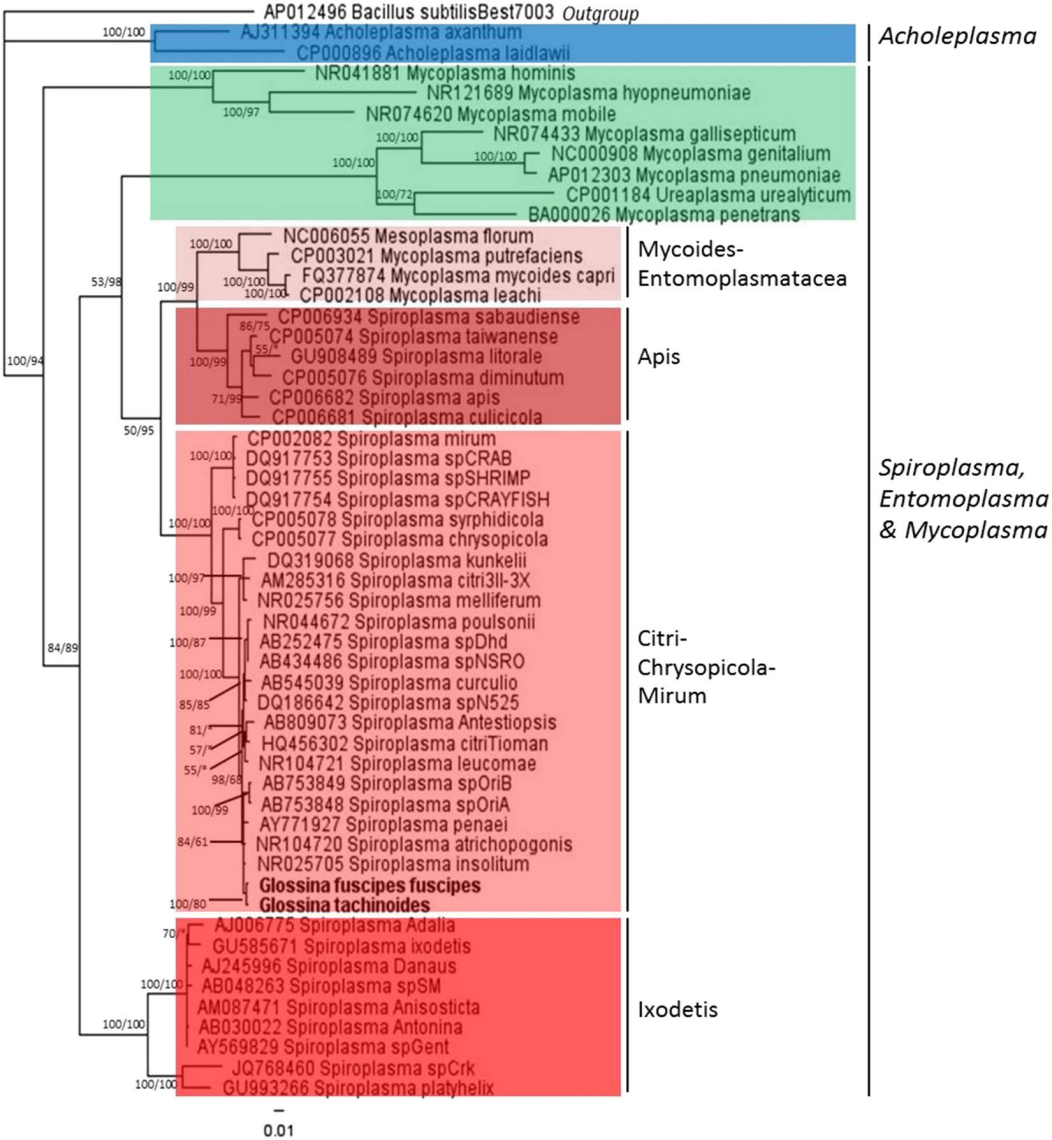
Bayesian inference phylogeny based on the 16S *rRNA* gene sequence: The topology resulting from the Maximum Likelihood (ML) method was similar. Bayesian posterior probabilities and ML bootstrap values based on 1000 replicates are given at each node, with the posterior probabilities given first followed by the ML bootstrap values (only values >50% are indicated), respectively. Asterisks indicate support values lower than 50%. The *Spiroplasma* strains present in *Gff* and *Gt* are indicated in bold letters. For each *Spiroplasma* species the GenBank accession number is given to the left of the name.

*Spiroplasma* strains from *Gff* flies of both sexes from laboratory colonies, a natural population from Uganda and from one natural population of *Gt* flies from Burkina Faso were genotyped by MLST analysis. Four laboratory and one field sample of *Gff* harbour *Spiroplasma* strains with identical sequences for all loci studied (Supplementary Table 4). Interestingly, the *Spiroplasma* strain present in *Gt* is distinct from the *Gff Spiroplasma* strain with sequence polymorphisms detected in all loci examined. Eight polymorphisms were observed in *fruR*, seven in the region 16S *rRNA*-23S *rRNA*-5S *rRNA*, four in 16S *rRNA*, three in *dna*A, two in *ftsZ*, and one in *rpoB* and *parE*. Both strains belong to the citri clade, which is mostly composed of plant pathogens (Fig. 4 and Supplementary Figures 2–7). Most of the pathogenic *Spiroplasma* species belong to the Citri clade^57^ with prominent examples including *S*. *kunkelii* that causes the corn stunt disease^21^, *S*. *phoeniceum* that infects periwinkle^58^, and *S*. *penaei* that infects Pacific white shrimp^42^. The closest relatives of the tsetse *Spiroplasma* strains are *S*. *insolitum* and *S*. *atrichopogonis*, which were isolated from a fall flower and a biting midge (Diptera: Ceratopogonidae) respectively^59, 60^. Neither *S*. *insolitum* or *S*. *atrichopogonis* have been reported to be pathogenic to plants or midges.

### *Spiroplasma* density across developmental stages

**Figure 5:**
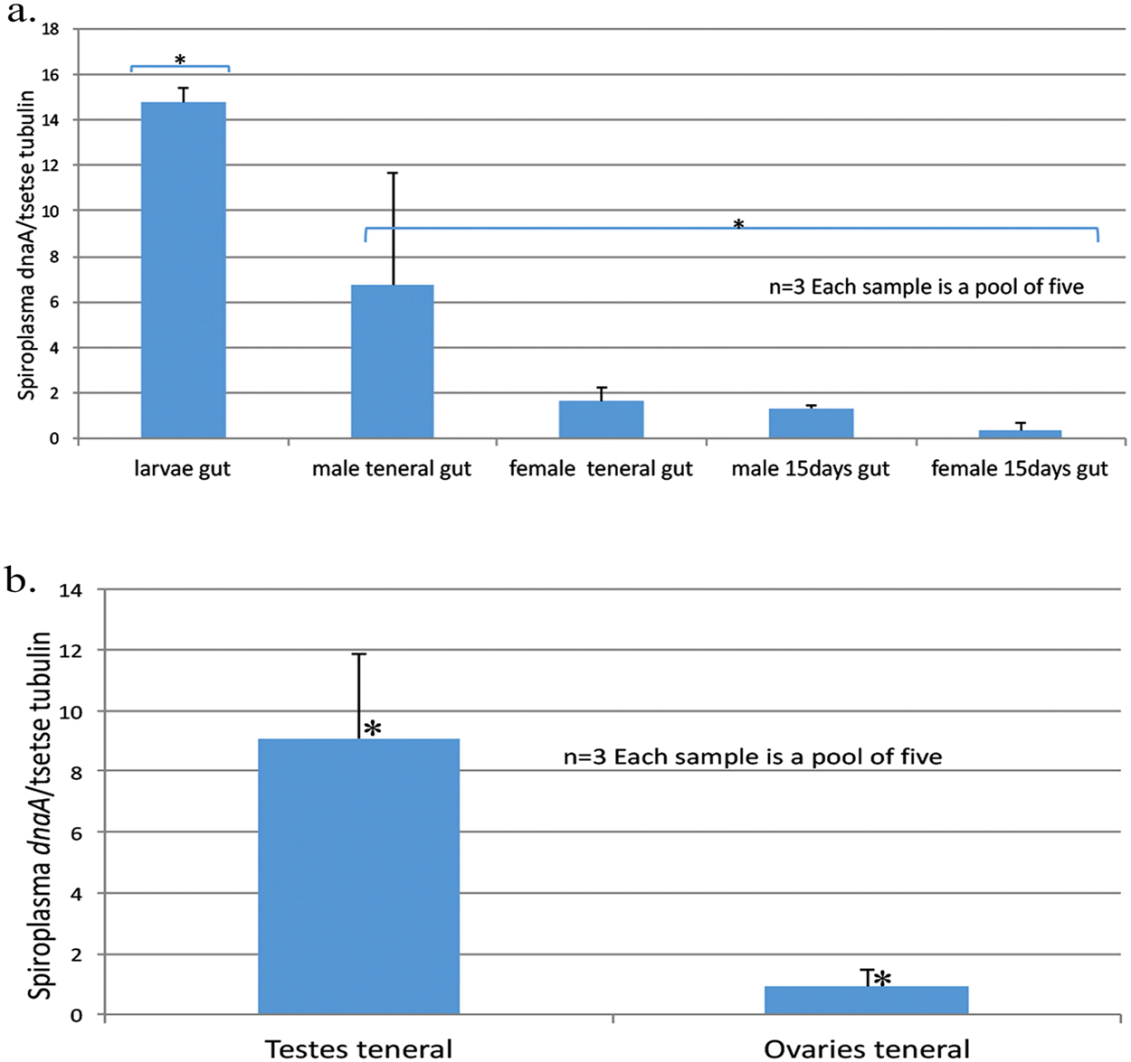
Quantification of *Spiroplasma* titre in terms of the symbiont *dnaA* gene copies normalized by the tsetse *β-tubulin* gene. (**a**) *Gff* gut from larvae, male and female teneral and 15-day old tsetse flies (n = 3, each sample is a poοl of five) p < 0.005, (**b**) gonads from male and female teneral tsetse flies (n = 3, each sample is a poοl of five), p < 0.05 (Anova test was performed; statistical significant differences are indicated with an asterisk*).

qPCR was used to assess the density of the *Spiroplasma* infection in larval guts, and in guts and gonads of males and females collected at two developmental stages: (a) teneral and (b) 15-day-old adults. *Spiroplasma* infection levels were significantly higher in larval guts compared to the guts of teneral or 15-day-old adults (Fig. 5a). There was no significant difference in the infection levels between testes of teneral and 15-day-old adults (Supplementary Figure 8). In a similar way no significant difference was observed between ovaries of teneral and 15-day-old adults (Supplementary Figure 9). However, there was a significant difference in *Spiroplasma* infection level between testes and ovaries from teneral flies (Fig. 5b).

**Figure 6:**
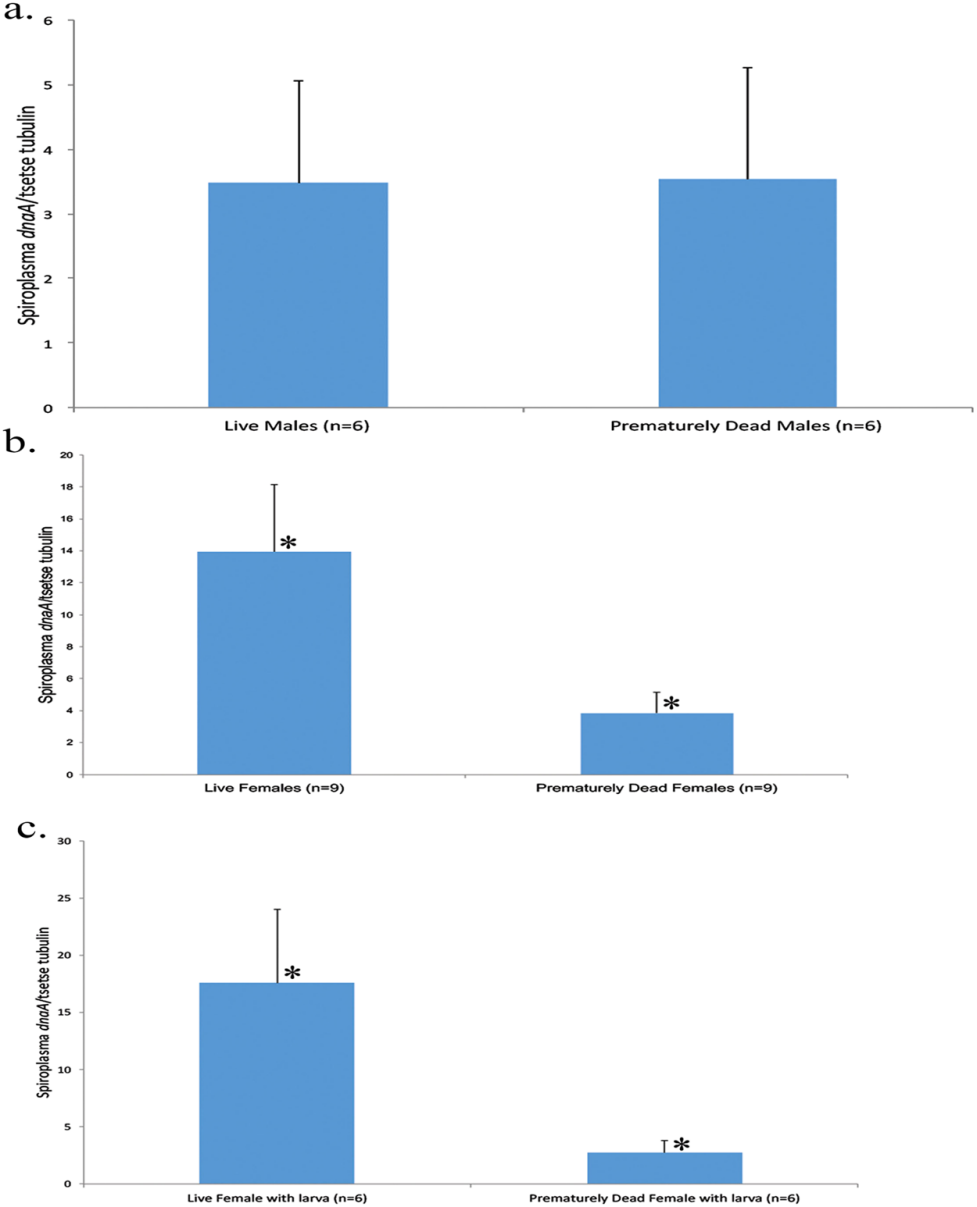
Quantification of *Spiroplasma* titre as *Spiroplasma dnaA* gene copy number normalized to the tsetse *β-tubulin* gene. (**a**) *Gff* whole insects from healthy/live males and prematurely dead males from the mass-rearing facility in Ethiopia (n = 6), (**b**) *Gff* whole insects from healthy/live females and prematurely dead females from the mass-rearing facility in Ethiopia (n = 9), p < 0.05. (**c**) *Gff* whole insects from healthy/live females carrying a larvae and prematurely dead females carrying a larva from the mass-rearing facility in Ethiopia (n = 6), p < 0.05. (ANOVA test was performed; statistical significant differences are indicated with an asterisk *).

*Spiroplasma* density was also examined in a mass-rearing colony where mortality was high and the colony was on the verge of collapse. Examination of live and dead insects indicated that in males *Spiroplasma* density was similar, whereas in females density was higher in live insects than in those that had recently perished (Fig. 6a and b). When we examined exclusively females carrying a larva, we found that the live females with a larva had a higher titre of *Spiroplasma* than gravid females that died prematurely (Fig. 6c). The prevalence of *Wolbachia*, *Arsenophonus*, *Cardinium*, and *Rickettsia* was also examined in whole tsetse flies from the collapsing colony. None of the 34 individuals tested were found to harbour any of the above mentioned symbionts.

### *In situ* hybridization of *Spiroplasma*

**Figure 7:**
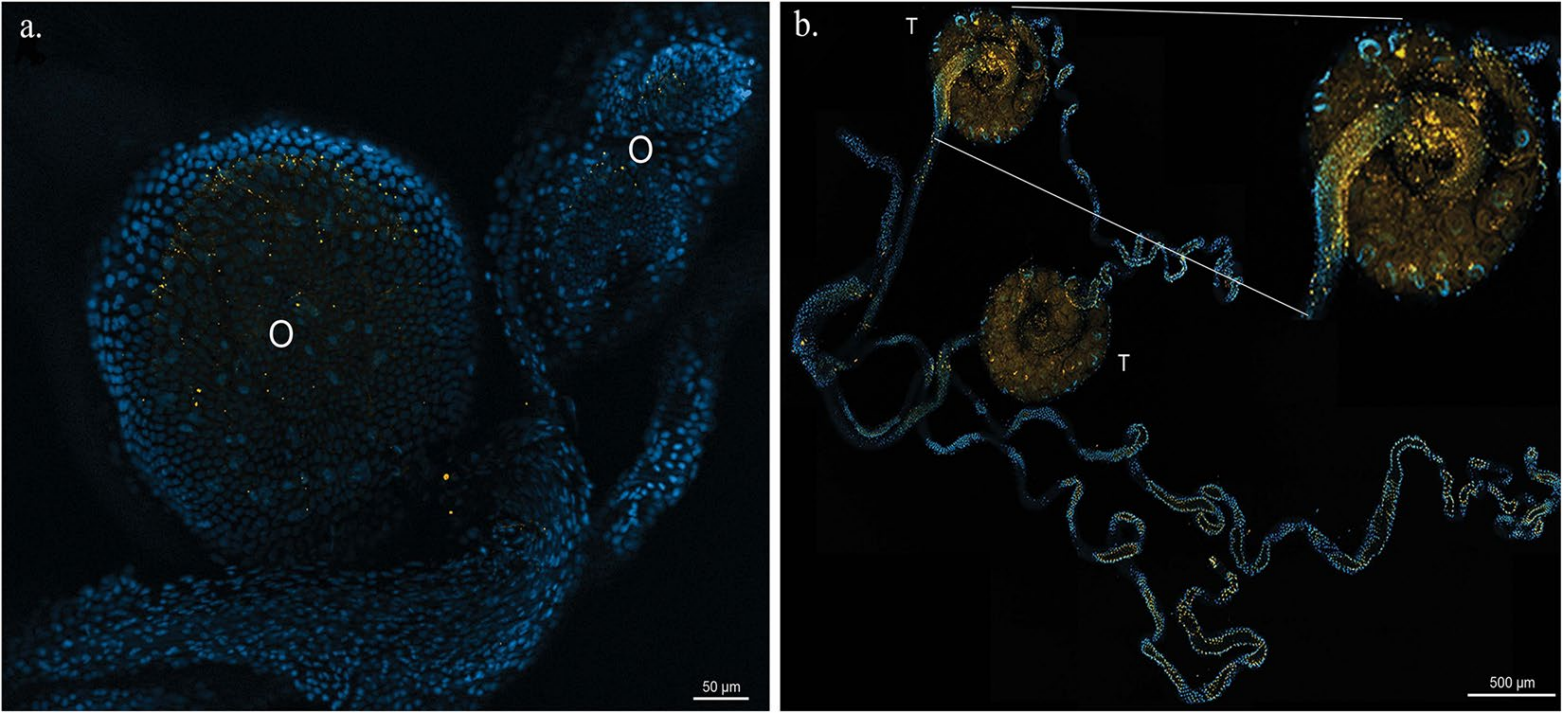
Localization of *Spiroplasma* in the male and female reproductive system of *Gff*. In fluorescent *in situ* hybridization (FISH) images blue and yellow indicate insect nuclear DNA and *Spiroplasma* respectively. (**a**) FISH on dissected ovaries (O), (**b**) FISH on dissected testes (T) with an inset showing a testis at a higher magnification.

Dissected ovaries and testes of teneral adults from a *Gff* laboratory colony were subjected to FISH using a *Spiroplasma* specific probe. *Spiroplasma* detection was sparse and sporadic in ovaries (Fig. 7a), while in testes it was observed at high densities (Fig. 7b).

## Discussion

The present study showed that the bacterial communities associated with tsetse flies are more complex than previously reported, thus challenging the *Wigglesworthia-Sodalis-Wolbachia* dogma^3, 61, 62^. Using 16S *rRNA* gene-based sequencing approaches, several additional bacterial genera with broad phylogenetic origins were discovered to be associated with the tsetse fly including *Klebsiella*, *Rickettsia* and *Spiroplasma*. The prevalence and infection levels observed in some tsetse species, particularly those of *Spiroplasma*, were similar to those seen for *Sodalis*, suggesting that they may play an important role in the biology and ecology of tsetse flies. The question is where these symbionts come from, and what factors determine the structure of the symbiotic communities of tsetse flies.

Previous studies have shown that the microbiota of tsetse flies is characterized by the presence of *Wigglesworthia*, *Sodalis* and *Wolbachia*. All three symbionts are maternally transmitted, while *Sodalis* can also be transmitted paternally, and colonize during the early juvenile stages: *Wigglesworthia* and *Sodalis* through milk gland secretions as larvae, and *Wolbachia* through the germ line during embryogenesis^3, 63, 64^. As larvae are intrauterine, the only bacteria that they encounter prior to pupation originate from within the adult female tsetse fly. Due to the obligate requirement of *Wigglesworthia*, there is high fidelity in vertical transmission from mother to offspring^65^. This makes it difficult for other bacteria to invade, as microbes occupy many of the available niches within the host from the early stages of development. Conversely, this also means that the tsetse immune system has evolved to accommodate bacteria, which could facilitate colonization by environmental microbes able to exploit deficits in the immune system. Due to the unique biology of tsetse flies, there is only a short time window for colonization between larval deposition and pupation in the soil. In addition, the colonizers would have to survive metamorphosis in order to persist.

Until recently, there was the notion that tsetse flies feed exclusively on blood, which is mostly sterile and therefore should not serve as a source of microbes. There is now evidence that *Gpg* flies deprived of a blood meal can feed on water or sugar water, and that sugar residues are detectable in wild-caught flies^66^. Therefore, it is possible that these previously unrecognized feeding habits could be a source of environmental microbes, and could be the origin of the low-frequency high-abundance infections observed in multiple individuals in this study.

*Spiroplasma* was detected in members of the *palpalis* sub-group (*Gff*, *Gpp* and *Gt*), whereas *Sodalis* was significantly more prevalent in *Gmed* (*fusca* group). Previous studies have also shown that *Sodalis* infection is more prevalent in *G*. *brevipalpis* (*fusca* group) than in *Gmm* and *Gpal* (both *morsitans* group)^67^. However, the relationship of *Spiroplasma* with the *palpalis* subgroup seems to be more exclusive than that of *Sodalis*, since the latter has previously been identified in individuals belonging to all tsetse sub-groups^18, 67, 68^.

A key approach to detecting invasive taxa is to sample whole insects rather than individual tissues such as the gut, where *Wigglesworthia* is dominant and will therefore obscure the detection of lower-abundance taxa. A broad phylogenetic range of host species is important to encompass the available diversity, as there seems to be variation between sub-groups, species, and even individuals within the same species.

For example, *Rickettsia* was discovered at high abundance in just one individual, despite the profiling of hundreds of insects by amplicon and PCR profiling. *Rickettsia* has been also identified in a previous study using an amplicon sequencing approach^18^ but also to *G*. *morsitans* from Senegal during a PCR screen^69^.

*Spiroplasma* infection was more prevalent in laboratory colonies with both males and females harbouring *Spiroplasma*, whereas in natural populations prevalence was lower and only females were infected. The lack of infection in wild individuals may be due to insufficient sampling effort, or could be due to the differences in population dynamics between laboratory-reared and wild-caught flies. It has been reported, for example, that some symbionts may be present in such low abundances that they are undetectable by conventional PCR screens^70^. MLST indicated that the strain found in wild *Gff* from Uganda was identical, based on the loci examined, to that in the colonized flies (originating from the Central African Republic), suggesting the association between *Spiroplasma* and *Gff* may be ancient. Although there have been no direct studies on the relative transmission rate of tsetse symbionts in the laboratory and field, paternal transmission during mating can occur for the secondary symbiont *Sodalis* ^64^. While this study only detected *Spiroplasma* infection in *palpalis* group flies, screening more specimens from the *morsitans* and *fusca* groups should provide more detailed information on the dynamics and spread of *Spiroplasma* infection in natural populations.

Another potential explanation for the absence of *Spiroplasma* in the *morsitans* and *fusca* groups is their frequent infection with *Wolbachia* ^12, 71^. In the *morsitans* group the prevalence of *Wolbachia* can vary between 9.5 and 100%, while in the *fusca* group it can vary from 0 to 15.6%^12, 71^. An existing *Wolbachia* infection may have led to the development of competitive exclusion with *Spiroplasma*, though it is not yet clear whether they share an ecological niche within the host, and whether co-occurrence could create evolutionary pressure strong enough to drive competitive exclusion^72^. In *D*. *melanogaster*, coinfections between *Wolbachia* and *Spiroplasma* were asymmetrical: *Spiroplasma* negatively affected the titre of *Wolbachia*, whereas *Wolbachia* density did not affect *Spiroplasma* titre^73^. Similarly to *Spiroplasma* in *Gff*, tissue tropism was observed in *D*. *melanogaster* infected with *Spiroplasma*, with the ovaries showing the highest density^73^. Competitive inter- and intraspecific microbial interactions have also been observed in mosquito vector species where mutual exclusion between *Asaia* and *Wolbachia* has been observed in the reproductive organs while native gut microbiota seems to prevent the vertical transmission of *Wolbachia* in *Anopheles* mosquitoes^74, 75^. *Gff* has previously been shown to harbor *Wolbachia*, though prevalence in natural populations is very heterogeneous, with an average infection rate of 44.3%^76^. *Spiroplasma*, on the other hand, is found at much lower frequency in natural populations, but is found at higher density per individual when compared with *Wolbachia*.

MLST analysis indicated that the *Spiroplasma* strains detected in *Gff* and *Gt* populations, albeit different, both belong to the citri clade. Prominent examples of taxa from this clade include *S*. *kunkelii*, *S*. *phoeniceum*, and *S*. *citri*, all of which are plant pathogens^21, 58, 77^. *S*. *poulsonii*, which has been shown to have a protective effect against parasitic wasps in *D*. *melanogaster*, is also a member of this clade^20^.

When examining gut tissues, *Spiroplasma* titre was highest in larvae, and gradually decreased in both males and females over the course of adulthood. High larval titre indicates vertical transmission from mother to offspring, possibly via the milk gland; a mechanism already exploited by *Wigglesworthia* and *Sodalis*. High larval density is an abnormal trait in the context of other insect-associated *Spiroplasma* species. Multiple strains of *Spiroplasma* infect a number of species of *Drosophila* and are able to induce a variety of phenotypes in their insect host ranging from parasitic reproductive manipulators to protective symbionts^20, 24, 78^. In *D*. *hydei* and *D*. *melanogaster*, *Spiroplasma* titre steadily increases during larval and adult development with no differentiation between males and females^73, 79^. Interestingly, *Drosophila* male killing *Spiroplasma* strains exhibit a very high titre in the haemolymph^78^, a pattern not observed in the *Gff Spiroplasma* strain (data not shown). In addition, *Spiroplasma* titre in *Gff* is much lower than that described for *Drosophila* male killing strains^29, 78^. *Wolbachia* is the only other maternally inherited endosymbiont found in *Drosophila*, and is also found in tsetse flies. *Wolbachia* confers density-dependent protection against insect viruses at different developmental stages in several *Drosophila* species^80–83^. Based on the above, it is possible that high *Spiroplasma* density may also play a role in larval fitness. This warrants further study, as protection against viral or bacterial pathogens during intrauterine larval development would constitute a rare phenotype for a bacterial endosymbiont. Recent studies in *D*. *melanogaster* showed that *Wolbachia* and *Spiroplasma* can affect immune signalling pathways in the presence of both insect pathogenic and non-pathogenic bacteria^84^.

Gut infection was maintained into adulthood, particularly in males. This suggests that *Spiroplasma* is either able to maintain infection during metamorphosis, possibly due to extracellular proliferation^73^, or that it can rapidly re-colonize upon reformation of the gut. *Spiroplasma* density was also significantly higher in the testes of teneral males than in the ovaries of teneral females. Localization to the testes suggests that *Spiroplasma* may be sexually transmitted from males to females, as has already been observed with *Sodalis* in tsetse flies, and *Asaia* in *Anopheles stephensi* ^64, 85^. The above properties can be exploited in paratransgenic approaches in a similar way to those currently being explored for *Sodalis* ^64, 86^ and *Asaia* ^87^.

In a collapsing colony of *Gff* flies, live females had a higher *Spiroplasma* density than prematurely dead females. This was true of both gravid and non-gravid females, and indicates that *Spiroplasma* may contribute to adult female fitness. It is therefore possible that *Spiroplasma* could play a protective role, as has been observed in other facultative strains of *Spiroplasma* ^20, 34, 88^ and/or a nutritional role.

## Materials and Methods

### Insect specimen collection and DNA isolation

All natural populations of *Glossina* specimens were collected in four countries, Burkina Faso, Uganda, United Republic of Tanzania, and South Africa (Table 1 and Supplementary Table 5). All wild flies were collected using biconical traps and collection intervals were four hours. Upon collection, flies were transferred to the main collection point and were placed in 100% acetone and stored at room temperature. Upon arrival in the lab, DNA was extracted immediately using the CTAB method (Cetyl trimethylammonium bromide)^89^. Laboratory populations were also analysed in a similar way. Samples of *Gff* suffering high mortality were collected from the mass rearing facility in Kality, Ethiopia. For a detailed description of the analysis performed see Supplementary Information.

### Multiplex Illumina MiSeq Sequencing, data, and statistical analysis

The V4 region of the 16 S *rRNA* gene was amplified using fusion primers F515 (5′-GTGCCAGCMGCCGCGGTAA-3′), and 805R (5′-GACTACCAGGGTATCTAAT-3′) from individual wild flies of *G*. *medicorum* (*Gmed*), *G*. *m*. *submorsitans* (*Gms*), *G*. *p*. *gambiensis* (*Gpg*), and *G*. *tachinoides* (*Gt*) collected in Burkina Faso. Data generated from the wild flies were combined with the data generated from 124 whole guts of *Gff*, *Gmm*, *Gpal* from a previous study^18^, which used an identical technical approach for amplicon generation and sequencing.

The V3-V4 region of the 16S *rRNA* gene was amplified using fusion primers U341F (5′-CCTACGGGRSGCAGCAG-3′), and 805 R (5′-GACTACCAGGGTATCTAAT-3′) from pools of tissues from larvae and adults of laboratory populations of *Gmm*, *Gff*, and *Gpal* (Supplementary Table 5).

For a detailed description of the PCR conditions please see Supplementary Information. The gene sequences reported in this study have been deposited in NCBI under Bioproject numbers PRJNA345319, and PRJNA345350-52. Statistical analyses was performed using Unifrac distances, PCoA analyses, CAP, ANOVA and Tukey-Kramer post-hoc tests as described in the Supplementary Information.

### PCR screening and *Spiroplasma* multi locus genotyping

*Gmm*, *Gff*, *Gpg*, and *Gpal* were assayed for the presence of *Spiroplasma*, *Arsenophonus*, *Cardinium*, and *Rickettsia* symbionts by PCR. An additional six species of Glossina (*G. austeni* (*Ga*), *G. brevipalpis* (*Gb*), *G. m. centralis* (*Gmc*), *Gms*, *G. p. palpalis* (*Gpp*) and *Gt* were screened for *Spiroplasma* only. The primer sequences used to detect each symbiont along with their target genes, product sizes, conditions, and annealing temperatures are listed in the Supplementary Information.

The *Spiroplasma* strains present in *Glossina* species were genotyped with a multi-locus sequence typing (MLST) approach using five marker genes (*rpo*B, *par*E, *dna*A, *fts*Z and *fru*R) and a 4,702 bp region spanning the 16S *rRNA*-23S *rRNA*-5S *rRNA* region. Details of the conditions used are presented in the Supplementary Information. Sequencing was performed as described previously^90^. All gene sequences generated in this study have been deposited into at GenBank under accession numbers KX159363-KX159393.

### Phylogenetic analysis

All nucleotide sequences were manually edited with Geneious 7.1.2. Multiple alignments were generated by MUSCLE^91^ and ClustalW^92^ by Geneious 7.1.2, and adjusted by eye. Phylogenetic analyses were conducted for all analysed *Spiroplasma* sequences (16 S *rRNA*, *rpoB*, *dnaA*, *parE*, *ftsZ* and *fruR* genes, and the region 16 S *rRNA*-23S *rRNA*-5S *rRNA* region) separately by two methods: Bayesian Inference (BI) and Maximum Likelihood (for a detailed description see Supplementary Information).

### Quantitative Real Time-PCR and Fluorescent *in situ* Hybridization (FISH)

*Spiroplasma* density was quantified by qPCR using the *dna*A *Spiroplasma* specific primers FqdnaA/RqdnaADoud for 35 cycles at 56 °C and normalized to the host *β-tubulin* gene. Primers and a detailed description used for the qPCR experiments are presented in Supplementary Table 6. qPCR data were analysed using a one-way ANOVA method, as described previously^93^ using the XLSTAT program.

*Gff* specimens from the Seibersdorf laboratory colony were used for FISH. Teneral male and female flies were dissected in PBS 2–3 days after eclosion. Dissected tissues were dried on poly-L-lysine-coated glass slides (Sigma, UK) for 20 min at 65 °C and kept at 4 °C until further use. Tissue samples were fixed in freshly prepared 4% paraformaldehyde solution for 30 min at 4 °C. A detailed description of tissue processing and image capture is included in the Supplementary Information.

## Electronic supplementary material

Supplementary Information
