# Supplementary material for: Challenging the Wigglesworthia, Sodalis, Wolbachia symbiosis dogma in tsetse flies: Spiroplasma is present in both laboratory and natural populations
Source: Sci Rep. 2017 Jul 5;7:4699. doi: 10.1038/s41598-017-04740-3 (PMC5498494; doi:10.1038/s41598-017-04740-3)
Supplement: Supplementary file 1 — Supplementary Information [file 41598_2017_4740_MOESM1_ESM.doc]

**Challenging the *Wigglesworthia*, *Sodalis*, *Wolbachia* symbiosis dogma in tsetse flies: *Spiroplasma* is present in both laboratory and natural populations**

Doudoumis V^1+^, Blow F^2+^, Saridaki A^1^, Augustinos A^1,3^, Dyer NA^4^, Goodhead I^2,#^, Solano P^5^, Rayaisse J-B^6^, Takac P^7,9^, Mekonnen S^8^, Parker AG^3^, Abd-Alla AMM^3^, Darby, A^2*^, Bourtzis K^3*^, Tsiamis G^1*^

**Supplementary Information**

**Supplementary Materials and Methods**

**Insect specimen collection and DNA isolation**

Laboratory populations from the Joint FAO/IAEA Insect Pest Control Laboratory (Seibersdorf), Laboratory of Epidemiology and Public Health (LEPH, Yale, US), Kenya Agriculture and Livestock Research Institute- Biotechnology Research Institute (KALRO – BRI in Nairobi, Kenya; former KARI-TRC), Centre international de recherche-développement sur l'élevage en zone subhumide (CIRDES in Bobo Dioulasso, Burkina Faso), Institute of Tropical Medicine (Antwerp, Belgium), Vector and Vector Borne Diseases Research Institute (Tanga, Tanzania), and the Slovak Academy of Sciences (Bratislava, Slovakia) have been used.

Tsetse fly tissues from laboratory reared flies were freshly dissected under sterile conditions and DNA extraction was performed as described previously^1^. In brief, tsetse flies (both adult and larvae) were surface sterilized prior to dissection, by dipping once in 70 % ethanol and once in 1X sterile PBS. Dissections were performed in 1X sterile PBS and samples were kept at -20 ^o^C after DNA extraction. Tissue collections included guts and reproductive organs (testes and ovaries). Guts were collected from third instar larvae and both teneral and 15 day-old males and females and reproductive tissues were collected. Samples were pooled from five individuals and three biological replicas were collected per sample. DNA was isolated using the Qiagen DNeasy kit (Qiagen, Valencia, CA). The quantity and quality of the DNA was measured using NanoDrop 1000 (Thermo Scientific).

**Multiplex Illumina MiSeq Sequencing, data, and statistical analysis**

Fusion primers U341F (5’-CCTACGGGRSGCAGCAG-3’), F515 (5’-GTGCCAGCMGCCGCGGTAA-3’), and 805R (5’-GACTACCAGGGTATCTAAT-3’) were used to amplify the V3-V4, and V4 region of the 16S *rRNA* gene. 805R reverse primers contained a unique Golay barcode specific to each sample for read de-multiplexing. Each PCR reaction was carried out in a volume of 25 μl containing 1 μl of template DNA, 12.5 μl NEBNext 2x High-Fidelity Master Mix (New England Biolabs, UK), 1 μl of each primer at 3mM concentration and 9.5 μl PCR-clean H_2_O. Cycling conditions were 30 seconds at 98^o^C, 25 cycles of 98^o^C for 10 seconds, 58^o^C for 30 seconds and 72^o^C for 30 seconds, followed by a final extension of 5 minutes at 72^o^C. Amplicons were cleaned using Ampure XP beads (Agencourt, UK), and re-suspended in 30 μl PCR-clean H_2_O. Products were quantified using the Qubit dsDNA High-Sensitivity assay (Life Technologies, UK), and an Agilent Bioanalyzer High-Sensitivity DNA chip (Agilent, UK). Samples were pooled at equimolar concentrations for size-selection by Pippin-Prep (Sage Science, UK), where a size range of 350-450 bp was extracted. Size-selected fragments underwent the same clean-up and quantification steps as above prior to sequencing. Sequencing was performed at the University of Liverpool Centre for Genomic Research, and at IMGM Laboratories GmbH on an Illumina MiSeq platform using 250 bp paired-end, and 300 bp paired-end read chemistry (Illumina, USA), respectively.

Raw sequencing reads were de-multiplexed and converted to FASTQ format using CASAVA version 1.8^2^. Cutadapt version 1.2.1 was used to trim Illumina adapter sequences from FASTQ files^3^. Reads were trimmed if 3bp or more of the 3’ end of a read matched the adapter sequence. Sickle version 1.200 was used to trim reads based on quality: any reads with a window quality score of less than 20, or which were less than 10 bp long after trimming, were discarded^4^. BayesHammer was used to correct reads based on quality^5^. Paired-end reads were assembled, trimmed by length and further corrected for error using PandaSeq^6^. Unassembled reads and reads outside the range of 240 to 260 bp once assembled were discarded. All subsequent analyses were conducted in QIIME version 1.7.0^7^. Sequences were clustered into Operational Taxonomic Units (OTUs) using USEARCH by de-novo OTU picking^8^. Chimeras were detected and omitted using the program UCHIME^9^ with the QIIME-compatible version of the SILVA 111 release database^10^. The most abundant sequence was chosen as the representative for each OTU. Taxonomy was assigned to representative sequences by BLAST^11^ against the SILVA 111 release database^10^. Representative sequences were aligned against the Greengenes core reference alignment^12^ using PyNAST^13^.

Between-sample diversity was calculated using weighted Unifrac distances, and PCoA analyses were performed on the resulting distance matrix. These calculations and those for alpha diversity were performed in QIIME version 1.7.0. ANOVA and Tukey-Kramer post-hoc tests were employed to detect statistical differences. Overall similarities in bacterial community structures were shown using the unconstrained ordination technique, principal coordinate analysis. Differences in community structure were viewed using the constrained ordination technique CAP, tested using the CAP classification success rate and CAP traceQ_m’HQ_m statistics, and were performed with 9999 permutations within PRIMER6+.

**PCR screening and *Spiroplasma* multi locus genotyping**

The PCR temperature profile was 95 °C for 5 min followed by 35 cycles of 95 °C for 30 sec, 30 sec at the appropriate annealing temperature for each pair of primers and the appropriate extension time for each amplicon at 72°C (Supplementary Table 2), and a final extension step of 72 °C for 10 min. PCR reactions were performed in 20 μl reaction mixtures containing 4 μl 5x reaction buffer (Promega, UK), 1.6 μl MgCl_2_ (25mM), 0.1 μl deoxynucleotide triphosphate mixture (25 mM each), 0.5 μl of each primer (25 μM), 0.1 μl of Taq (Promega 1U/μl), 12.2 μl water and 1 μl of template DNA. All host DNA samples were positive for PCR amplification using the 12S *rRNA* gene arthropod universal primers, indicating satisfactory DNA quality.

*Spiroplasma* strains present in *Glossina* species were genotyped with a multi-locus sequence approach using five marker genes (*rpo*B*, par*E*, dna*A*, fts*Z and *fru*R) and a 4,702 bp region spanning the 16S *rRNA*-23S *rRNA*-5S *rRNA* region. Primers and PCR conditions used are presented in Supplementary Table 2. Briefly, 5 min of denaturation at 95 °C preceded thirty-five PCR cycles of 30 sec at 95 °C, 30 sec at the appropriate temperature for each pair of primers and the appropriate extension time for each amplicon at 72 °C, followed by a final extension step at 72 °C for 10min. PCR reactions were carried out in 25 μl reaction mixtures containing 12.5 μl High Fidelity Ready Mix Reaction Buffer (Promega, UK), 0.3 μl of each primer (25 μM), 10.7 μl water and 1.2 μl of template DNA. The PCR products were purified by a PEG (Polyethylene glycol) - NaCl method as described previously^14^.

**Phylogenetic analysis**

Best-fit models of nucleotide substitution for constructing phylogenies of our data sets were estimated using the Model Test and Akaike Information Criterion^15^ by PAUP version 4.0b10^16^. For six out of seven analysed *Spiroplasma* alignments (16S *rRNA,* region 16S-23S-5S *rRNA, rpoB, dnaA, parE,* and *ftsZ*), the submodel GTR+I+G was selected, while the model K81uf+I was selected for the gene *fruR*. Bootstrap values were obtained with 1,000 replications. Maximum-Likelihood trees were constructed by using MEGA version 5.2.2 while Bayesian phylogenetic analyses were performed using MrBayes 3.1^17^ as implemented in Geneious 7.1.2^18^. Run parameters included four Markov chain Monte Carlo (MCMC) chains with one million generations, sampled every 100 generations, and with the first 10,000 trees discarded as burn-in. Posterior probabilities were computed for the remaining trees.

**qPCR (quantitative Real Time-PCR) and Fluorescent *In Situ* Hybridization (FISH)**

*Spiroplasma* qPCR was performed using the *dnaA* *Spiroplasma* specific primers FqdnaA/RqdnaADoud for 35 cycles at 56 °C and normalized to the host *β-tubulin* gene, which was amplified using primer set GmmtubqF/GmmtubqR for 35 cycles at 56 °C. The primers used for the qPCR experiments are presented in Supplementary Table 2. Reactions were performed using the KAPA SYBR FAST qPCR Kit (KAPA Biosystems, UK) in 10 μl (2 replicates for each reaction) containing: 5 μl 2x KAPA SYBR FAST qPCR Master Mix Universal, 0.08 μl from each primer (25 μM), 4.34 μl water and 0.5 μl total DNA (~ 20ng). Real-time PCR runs were conducted in MJ Research Opticon 2 (MJ Research, USA). Amplifications were carried out in a 96-well plate and each biological sample had a minimum of three replicates. Symbiont density (relative density) was defined as the copy number of the *dnaA* *Spiroplasma* specific fragment relative to the copy number of the *β-tubulin* host fragment^19^. Statistical significance was determined using ANOVA. Internal standard curves were generated for each primer set by cloning the amplicon into a pGEM-Teasy vector (Promega) according to the manufacturer’s instructions. All assays were carried out in duplicate, and replicates were averaged for each sample. Negative controls were included in all amplification reactions.

For the FISH experiments after fixation, slides were rinsed twice with PBS. To permeabilize the membranes, slides were coated at 60 °C with a few drops of 70% acetic acid, rinsed with PBS after 1 min incubation, dehydrated through a graded ethanol series and left on the bench until completely dry. Then, slides were incubated in 0.2% TritonX-100 in PBS for 2 min at room temperature, rinsed with PBS, dehydrated in ethanol and air-dried. Pre-hybridization occurred for 15 min in the pre-hybrization buffer, containing 20 mM Tris-HCl pH 8.0, 0.9 N NaCl, 0.01% sodium dodecyl sulphate and 30% formamide. The hybridization step was performed at 35 °C overnight by coating slides with 100 μl of prehybridization buffer supplemented with 1 μg/ml Cy3-labelled Spr403 probe targeting *Spiroplasma*^20^. From this step on, all slides were kept in the dark. After hybridization, slides were rinsed in prehybridization buffer twice at 42 °C for 30 min each, followed by a quick rinse in PBS and in deionized water. Then, the slides were air-dried, mounted with VECTASHIELD® mounting medium containing 1.5 μg/ml DAPI (Vector Laboratories, UK) and kept in the dark at 4 °C until observation on an Axio Observer.Z1 inverted microscope (Zeiss, Germany). Images were captured using an ApoTome.2 imaging system for optical sectioning, AxioCam MRm camera and Zen imaging software (Zeiss, Germany).

**References for Supplementary Information**

1. Augustinos, A. A. et al. Exploitation of the medfly gut microbiota for the enhancement of Sterile Insect Technique: use of *Enterobacter* sp. in larval diet-based probiotic applications. *PLoS ONE* **10**, e0136459 (2015).
2. Illumina. CASAVA v1.8 User Guide (2011).
3. Martin M. Cutadapt removes adapter sequences from high-throughput sequencing reads. *EMBnet.journal* **17**, 10–12 (2011).
4. Joshi, N. & Fass, J. Sickle: A sliding-window, adaptive, quality-based trimming tool for FastQ files. http://github.com/najoshi/sickle (Accessed February 1, 2016) (2011).
5. Nikolenko, S.I., Korobeynikov, A.I. & Alekseyev, M.A. BayesHammer: Bayesian clustering for error correction in single-cell sequencing. *BMC Genomics* **14**, 1–11 (2013).
6. Masella, A.P., Bartram, A.K., Truszkowski, J.M., Brown, D.G. & Neufeld, J.D. PANDAseq: paired-end assembler for illumina sequences. *BMC Bioinformatics* **13**, 31 (2012).
7. Caporaso, J.G. et al. QIIME allows analysis of high-throughput community sequencing data. *Nat. Methods* **7**, 335–336 (2010).
8. Edgar, R.C. Search and clustering orders of magnitude faster than BLAST. *Bioinformatics* **26**, 2460–2461 (2010).
9. Edgar, R.C., Haas, B.J., Clemente, J.C., Quince, C. & Knight, R. UCHIME improves sensitivity and speed of chimera detection. *Bioinformatics* **27**, 2194–2200 (2011).
10. Quast, C. et al. The SILVA ribosomal RNA gene database project: improved data processing and web-based tools. *Nucleic Acids Res.* **41**, D590–D596 (2013).
11. Altschul, S.F., Gish, W., Miller, W., Myers, E.W. & Lipman, D.J. Basic local alignment search tool. *J. Mol. Biol.* **215**, 403–410 (1990).
12. DeSantis, T.Z. et al. Greengenes, a chimera-checked 16S rRNA gene database and workbench compatible with ARB. *Appl. Environ. Microbiol.* **72**, 5069–72 (2006).
13. Caporaso, J.G. et al. PyNAST: a flexible tool for aligning sequences to a template alignment. *Bioinformatics* **26,** 266–267 (2010).
14. Tsiamis, G. et al. Olive-mill wastewater bacterial communities display a cultivar specific profile. *Curr. Microbiol.* **64**, 197–203 (2012).
15. Akaike, H. Autoregressive model fitting for control. *Ann. Inst. Stat. Math.* **23**, 163–180 (1971).
16. Swofford, D. L. PAUP*, Version 4.0 b4a. Sinauer (2000).
17. Ronquist, F. & Huelsenbeck, J.P. MrBayes 3: Bayesian phylogenetic inference under mixed models. *Bioinformatics* **19**, 1572–1574 (2003).
18. Kearse, M. et al. Geneious Basic: An integrated and extendable desktop software platform for the organization and analysis of sequence data. *Bioinformatics* **28**, 1647–1649 (2012).
19. Alam, U. et al. Implications of microfauna-host interactions for trypanosome transmission dynamics in *Glossina fuscipes fuscipes* in Uganda. *Appl. Environ. Microbiol.* **78**, 4627–4637 (2012).
20. Matsuura, Y. Bacterial symbionts of a devastating coffee plant pest, the stinkbug *Antestiopsis thunbergii* (Hemiptera: Pentatomidae). *Appl. Environ. Microbiol.* **80**, 3769–3775 (2014).
21. Aksoy, E. et al. Analysis of multiple tsetse fly populations in Uganda reveals limited diversity and species-specific gut microbiota. *Appl. Environ. Microbiol.* **80**, 4301–4312 (2014).
22. Hanner, R. & Fugate, M. Branchiopod phylogenetic reconstruction from 12S rDNA sequence data. *J. Crustacean Biol.* **17**, 174–183 (1997).
23. Mateos, M. et al. Heritable endosymbionts of *Drosophila*. *Genetics* **174**, 363–376 (2006).
24. Fukatsu, T. & Nikoh, N. Endosymbiotic microbiota of the bamboo Pseudococcid *Antonina crawii* (Insecta, Homoptera). *Appl. Environ. Microbiol.* **66**, 643–650 (2000).
25. Fukatsu, T. & Nikoh, N. Two intracellular symbiotic bacteria from the Mulberry Psyllid *Anomoneura mori* (Insecta, Homoptera). *Appl. Environ. Microbiol.* **64**, 3599–3606 (1998).
26. Fukatsu, T., Tsuchida, T., Nikoh, N. & Koga R. *Spiroplasma* Symbiont of the Pea Aphid, *Acyrthosiphon pisum* (Insecta: Homoptera). *Appl. Environ. Microbiol.* **67**, 1284–1291 (2001).
27. Gotoh, T., Noda, H. & Ito, S. *Cardinium* symbionts cause cytoplasmic incompatibility in spider mites. *Heredity* **98**, 13–20 (2006).
28. Weeks, A. R., Velten, R. & Stouthamer R. Incidence of a new sex-ratio-distorting endosymbiotic bacterium among arthropods. *Proc. Biol. Sci.* **270**, 1857–1865 (2003).
29. Duron, O. et al. The diversity of reproductive parasites among arthropods: *Wolbachia* do not walk alone. *BMC Biol.* **6**, 27 (2008).
30. Haselkorn, T.S., Markow, T. A. & Moran, N. A. Multiple introductions of the *Spiroplasma* bacterial endosymbiont into Drosophila. *Mol. Ecol.* **18**, 1294–1305 (2009).
31. Guz, N., Attardo, G. M., Wu, Y. & Aksoy S. Molecular aspects of transferrin expression in the tsetse fly (*Glossina morsitans morsitans*). *J. Insect Physiol.* **53,** 715–723 (2007).
32. Harumoto, T., Anbutsu, H. & Fukatsu, T. Male-Killing Spiroplasma induces sex-specific cell death via host apoptotic pathway. *PLoS Pathog.* **10**, e1003956 (2014).
33. Bi, K., Huang, H., Gu, W., Wang, J. & Wang W. Phylogenetic analysis of Spiroplasmas from three freshwater crustaceans (*Eriocheir sinensis*, *Procambarus clarkia* and *Penaeus vannamei*) in China. *J. Invertebr. Pathol.* **99,** 57–65 (2008).
34. Heres, A. & Lightner, D. V. Phylogenetic analysis of the pathogenic bacteria *Spiroplasma* *penaei* based on multilocus sequence analysis. *J. Invertebr. Pathol.* **103**: 30–35 (2010).
35. Montenegro, H., Solferini, V. N., Klaczko, L.B. & Hurst, G.D.D. Male-killing *Spiroplasma* naturally infecting *Drosophila melanogaster*. *Insect Mol. Biol.* **14**: 281–287 (2005).
36. Anbutsu, H. & Fukatsu, T. Population dynamics of male-killing and non-male-killing Spiroplasmas in *Drosophila melanogaster*. *Appl. Environ. Microbiol.* **69**, 1428–1434 (2003).

**Supplementary Tables**

**Supplementary Table 1** Richness and diversity estimation of the 16S rRNA libraries from the amplicon sequence analysis of the natural populations

|  |  | **Species richness indices** | | **Species diversity indices** | |
| --- | --- | --- | --- | --- | --- |
| **Samples** | **Number of OTUs** | **Chao1** | **ACE** | **Shannon** | **Simpson** |
| *Gff* Busime Female Gut | 106.66±20.70 | 158.07±35.08 | 159.47± 30.17 | 0.60±0.07 | 0.88±0.02 |
| *Gff* Busime Male Gut | 130.25±16.68 | 182.59±27.12 | 187.56± 33.36 | 0.67±0.08 | 0.86±0.02 |
| *Gff* Dokolo Female Gut | 146.57±33.61 | 202.42±47.58 | 200.96±41.47 | 0.61±0.11 | 0.87±0.03 |
| *Gff* Dokolo Male Gut | 180.83±28.05 | 258.40±46.40 | 249.66±50.22 | 0.68±0.08 | 0.86±0.02 |
| *Gff* Kaberamaido Female Gut | 143.27±46.44 | 199.10±72.14 | 204.39±64.26 | 0.66±0.22 | 0.87±0.04 |
| *Gff* Kaberamaido Male Gut | 160.79±50.70 | 233.86±68.19 | 225.75±56.82 | 0.64±0.10 | 0.87±0.03 |
| *Gff* Murchison Falls Female Gut | 132±16.97 | 183.79±10.40 | 181.69±12.12 | 0.53±0.09 | 0.89±0.02 |
| *Gff* Murchison Falls Male Gut | 142.55±30.25 | 207.82±33.19 | 197.31±28.34 | 0.56±0.10 | 0.88±0.02 |
| *Gff* Otuboi Female Gut | 137.25±31.76 | 202.96±39.03 | 201.71±34.21 | 0.54±0.08 | 0.89±0.02 |
| *Gff* Otuboi Male Gut | 146±34.11 | 219.55±58.64 | 214.50±46.68 | 0.61±0.10 | 0.87±0.02 |
| *Gmed* Burkina Faso Female [WI] | 58.43±70.47 | 94.41±109.87 | 100.84±108.9 | 1.27±0.37 | 0.68±0.11 |
| *Gmed* Burkina Faso Male [WI] | 41±15.54 | 55.66±18.36 | 64.87±25.47 | 1.23±0.16 | 0.72±0.05 |
| *Gmm* Murchison Falls Female Gut | 78.5±7.94 | 115.48± 28.47 | 124.98±29.09 | 0.21±0.04 | 0.97±0.008 |
| *Gmm* Murchison Falls Male Gut | 97±28.28 | 128.53±44.58 | 136.91±48.21 | 0.24±0.04 | 0.96±0.006 |
| *Gms* Murchison [WI] | 99.63±152.44 | 139.48±172.48 | 143.16±165.8 | 1.13±0.25 | 0.71±0.10 |
| *Gpal* Murchison Falls Female Gut | 92.5±17.72 | 123.1±21.71 | 123.43±18.29 | 0.68±0.54 | 0.83±0.14 |
| *Gpal* Murchison Falls Male Gut | 101.4±19.73 | 124.75±27.48 | 126.74±23.13 | 0.91±0.94 | 0.78±0.23 |
| *Gpg* Burkina Faso [WI] | 393.88±41.79 | 494.73±40.72 | 491.31±41.21 | 0.83±0.27 | 0.79±0.11 |
| *Gt* Burkina Faso Female [WI] | 393.4±65.3 | 480.7±70.4 | 472.52±59.85 | 0.59±0.26 | 0.88±0.09 |

*Gmm*: *Glossina morsitans morsitans; Gff*: *Glossina fuscipes fuscipes; Gpal*: *Glossina pallidipes*; *Gmed: Glossina medicorum; Gms: Glossina morsitants submorsitans*; *Gpg*: *Glossina palpalis gambiensis*; *Gt: Glossina tachinoides.* WI: whole insect

**Supplementary Table 2** Mean relative abundance of *Wolbachia* and *Spiroplasma* in larvae, guts and reproductive tissues of teneral adults, and guts and reproductive tissues of 15d post-eclosion adults in three lab populations of tsetse fly (*Gff:* *G. f. fuscipes, Gmm: G. m. morsitans,* and *Gpal:* *G. pallidipes*).

| **Species (no. individuals)** | ***Wolbachia* mean relative abundance and S.D.** | ***Spiroplasma* mean relative abundance and S.D.** |
| --- | --- | --- |
| *Gff* (24) | 0.03 ± 0.01 | 2.74 ± 1.05 |
| *Gmm* (27) | 1.65 ± 0.36 | <0.01 ± <0.01 |
| *Gpal* (27) | 0.23 ± 0.11 | <0.00 ± <0.01 |

**Supplementary Table 3** Richness and diversity estimation of the 16S rRNA libraries from the amplicon sequence analysis of the lab populations

|  |  | **Species richness indices** | | **Species diversity indices** | |
| --- | --- | --- | --- | --- | --- |
| **Samples** | **Number of OTUs** | **Chao1** | **ACE** | **Shannon** | **Simpson** |
| *Gmm* larvae | 139.0±36.0 | 84.4±30.7 | 90.9±31.5 | 1.73±0.23 | 0.64±0.08 |
| *Gmm* gut male 1d | 74.0±10.8 | 55.6±9.5 | 64.8±13.3 | 1.38±0.28 | 0.55±0.13 |
| *Gmm* testes 1d | 89.7±12.3 | 77.5±14.1 | 82.0±11.5 | 1.45±0.30 | 0.54±0.12 |
| *Gmm* gut female 1d | 53.3±6.9 | 32.8±5.6 | 35.5±6.4 | 1.14±0.15 | 0.44±0.07 |
| *Gmm* ovaries 1d | 121.0±16.3 | 86.8±15.0 | 92.3±6.3 | 1.57±0.38 | 0.53±0.11 |
| *Gmm* gut male 15d | 57.7±6.7 | 41.1±9.4 | 44.4±10.1 | 1.46±0.18 | 0.59±0.09 |
| *Gmm* testes 15d | 132.3±14.9 | 91.1±18.6 | 99.4±19.5 | 1.05±0.10 | 0.37±0.03 |
| *Gmm* gut female 15d | 55.3±1.5 | 48.3±13.2 | 40.5±5.0 | 1.32±0.12 | 0.53±0.05 |
| *Gmm* ovaries 15d | 70.3±18.6 | 56.1±9.0 | 59.6±11.7 | 1.05±0.07 | 0.39±0.01 |
| *Gff* larvae | 63.7±1.8 | 64.9±8.2 | 62.1±12.5 | 1.45±0.26 | 0.59±0.11 |
| *Gff* gut male 1d | 39.3±1.7 | 33.6±2.7 | 33.3±1.0 | 0.97±0.06 | 0.37±0.03 |
| *Gff* testes 1d | 152.7±47.9 | 86.2±20.0 | 88.3±20.5 | 1.55±0.24 | 0.58±0.11 |
| *Gff* gut female 1d | 40.7±2.6 | 27.8±0.3 | 29.1±0.3 | 1.18±0.14 | 0.47±0.07 |
| *Gff* ovaries 1d | 51.37±3.50 | 67.02±6.32 | 68.54±6.27 | 3.02±0.1 | 0.8±0.01 |
| *Gff* gut male 15d | 45.0±4.7 | 26.2±1.2 | 26.1±0.9 | 1.48±0.01 | 0.61±0.00 |

| *Gff* testes 15d | 75.0±19.0 | 82.6±22.3 | 73.6±17.1 | 1.16±0.13 | 0.43±0.07 |
| --- | --- | --- | --- | --- | --- |
| *Gff* gut female 15d | 41.7±3.5 | 42.5±8.3 | 34.2±3.3 | 1.39±0.08 | 0.58±0.04 |
| *Gff* ovaries 15d | 103.3±12.2 | 95.3±15.3 | 92.1±10.9 | 0.95±0.07 | 0.35±0.02 |
| *Gpal* larvae | 92.7±20.7 | 63.7±22.1 | 59.1±12.5 | 0.98±0.06 | 0.37±0.02 |
| *Gpal* gut male 1d | 46.7±6.2 | 18.4±0.5 | 19.5±0.4 | 0.75±0.03 | 0.34±0.01 |
| *Gpal* testes 1d | 120.5±13.5 | 104.3±2.7 | 98.7±0.4 | 2.00±0.53 | 0.64±0.17 |
| *Gpal* gut female 1d | 32.5±2.9 | 21.6±4.0 | 21.7±3.3 | 0.69±0.00 | 0.35±0.01 |
| *Gpal* ovaries 1d | 309.7±21.2 | 180.4±16.9 | 192.9±11.5 | 2.62±0.08 | 0.82±0.01 |
| *Gpal* gut male 16d | 42.3±14.5 | 28.2±2.9 | 29.8±1.7 | 1.36±0.15 | 0.54±0.06 |
| *Gpal* testes 16d | 155.7±12.3 | 102.8±14.6 | 116.9±22.5 | 1.61±0.10 | 0.55±0.04 |
| *Gpal* gut female 16d | 43.7±6.7 | 29.0±4.1 | 29.1±3.0 | 1.24±0.08 | 0.52±0.02 |
| *Gpal* ovaries 16d | 131.7±6.5 | 93.7±11.2 | 100.5±10.5 | 1.59±0.09 | 0.57±0.04 |

*Gmm*: *Glossina morsitans morsitans; Gff*: *Glossina fuscipes fuscipes; Gpal*: *Glossina pallidipes*

**Supplementary Table 4.** *Spiroplasma* MLST genotyping in *Glossina* species

| Origin (Area, Collection Date, Population, sex, tissue) | *Spiroplasma* MLST | | | | | | |
| --- | --- | --- | --- | --- | --- | --- | --- |
|  | *16S rRNA* | *16S rRNA-23S rRNA-5S rRNA* | *rpoB* | *dnaA* | *parE* | *fruR* | *ftsZ* |
| *G. f. fuscipes* Bratislava lab-colony ♀ | Allele 1 | Allele2 | Allele3 | Allele4 | Allele5 | Allele6 | Allele7 |
| *G. f. fuscipes* Bratislava lab-colony ♂ | Allele 1 | Allele2 | Allele3 | Allele4 | Allele5 | Allele6 | Allele7 |
| *G. f. fuscipes* Uganda ♀ | Allele 1 | Allele2 | Allele3 | Allele4 | Allele5 | Allele6 | Allele7 |
| *G. f. fuscipes* Seibersdorf lab-colony ♀ | Allele 1 | Allele2 | Allele3 | Allele4 | Allele5 | Allele6 | Allele7 |
| *G. f. fuscipes* Seibersdorf lab-colony ♂ | Allele 1 | Allele2 | Allele3 | Allele4 | Allele5 | Allele6 | Allele7 |
| *G. tachinoides* Burkina Faso ♀ | Allele 8 (4) | Allele9  (7) | Allelel0 (2) | Allele11 (1) | Allele12 (8) | Allele13 (1) | Allele14 (1) |

Number in brackets indicate the polymorphisms observed

**Supplementary Table 5** Metadata for all samples profiled by Illumina MiSeq amplicon sequencing.

| **Species** | **Population Type / Reference / Year of collection** | **Location** | **Tissue** | **No. of Samples** | **No. of Individuals per Sample** |
| --- | --- | --- | --- | --- | --- |
| ***G. f. fuscipes*** | Field^21^ / 2014 | Uganda (Busime) | Gut | 13 | 1 |
|  | Field^21^ / 2014 | Uganda (Dokolo) | Gut | 10 | 1 |
|  | Field^21^ / 2014 | Uganda (Kaberamaido) | Gut | 25 | 1 |
|  | Field^21^ / 2014 | Uganda (Murchison Falls) | Gut | 10 | 1 |
|  | Field^21^ / 2014 | Uganda (Otubio) | Gut | 13 | 1 |
|  | Lab / This study / 2013 | Lab colony^1^ | Larval gut | 3 | 5 |
|  | Lab / This study / 2013 | Lab colony^1^ | Gut 1d | 6^ | 5 |
|  | Lab / This study / 2013 | Lab colony^1^ | Gut 15d | 6^ | 5 |
|  | Lab / This study / 2013 | Lab colony^1^ | Reproductive 1d | 6^ | 5 |
|  | Lab / This study / 2013 | Lab colony^1^ | Reproductive 15d | 6^ | 5 |
| ***G. medicorum*** | Field / This study /2010 | Burkina Faso (Folonzo) | Whole | 8 | 1 |
| ***G. m. morsitans*** | Field^21^ / 2014 | Uganda (Murchison Falls) | Gut | 6 | 1 |
|  | Lab / This study / 2013 | Lab colony^1^ | Larval gut | 3 | 5 |
|  | Lab / This study / 2013 | Lab colony^1^ | Gut 1d | 6^ | 5 |
|  | Lab / This study / 2013 | Lab colony^1^ | Gut 15d | 6^ | 5 |
|  | Lab / This study / 2013 | Lab colony^1^ | Reproductive 1d | 6^ | 5 |
|  | Lab / This study / 2013 | Lab colony^1^ | Reproductive 15d | 6^ | 5 |
| ***G. m. submorsitans*** | Field* / This study /2010 | Burkina Faso (Folonzo) | Whole | 8 | 1 |
| ***G. pallidipes*** | Field^21^ / 2014 | Uganda (Murchison Falls) | Gut | 42 | 1 |
|  | Field / This study /2010 | Burkina Faso (Folonzo) | Whole | 27 | 1 |
|  | Lab / This study / 2013 | Lab colony^1^ | Larval gut | 3 | 5 |
|  | Lab / This study / 2013 | Lab colony^1^ | Gut 1d | 6^ | 5 |
|  | Lab / This study / 2013 | Lab colony^1^ | Gut 15d | 6^ | 5 |
|  | Lab / This study / 2013 | Lab colony^1^ | Reproductive 1d | 6^ | 5 |
|  | Lab / This study / 2013 | Lab colony^1^ | Reproductive 15d | 6^ | 5 |
| ***G. p. gambiensis*** | Field / This study /2010 | Burkina Faso (Folonzo) | Whole | 8 | 1 |
| ***G. tachinoides*** | Field*# / This study /2010 | Burkina Faso (Folonzo) | Whole | 8 | 1 |

(^ = 3 male-only samples and 3 female-only samples. * = samples analysed by PCR and amplicon profiling. # = population used for MLST analysis. ^1^Joint FAO/IAEA Insect Pest Control Laboratory (Seibersdorf)

**Supplementary Table 6.** Primers used for PCR, qPCR and sequencing (SR) reactions

| **Name / Reference** | **Primer Sequence 5’-3’** | **Gene region** | **Annealing temperature / extension time** | **Fragment size (bp)** | **Primer type** |
| --- | --- | --- | --- | --- | --- |
| 12SCFR^22^  12SCRR^22^ | GAG AGT GAC GGG CGA TAT GT  AAA CCA GGA TTA GAT ACC CTA TTA T | *mt 12S rRNA*  *host* | 54 ^o^C / 1min | 377 | PCR |
| 63F^23^  TKSSsp^24^ | GCCTAATACATGCAAGTCGAAC  TAGCCGTGGCTTTCTGGTAA | *16S rRNA*  *Spiroplasma* | 59 ^o^C / 1min | 455 | PCR, SR |
| 16SA1^25^  Rick 16SR^26^ | AGAGTTTGATCTGGCTCAG  CATCCATCAGCGATAAATCTTTC | *16S rRNA Rickettsia* | 55 ºC / 1 min | 200 | PCR |
| CLO f1^27^  CLO r1^27^ | GGAACCTTACCTGGGCTAGAATGTATT  GCCACTGTCTTCAAGCTCTACCAAC | *16S rRNA Cardinium* | 56 ºC / 1:30min | 466 | PCR |
| CLOF^28^  CLOR^28^ | GCGGTGTAAAATGAGCGTG  ACCTMTTCTTAACTCAAGCCT | *16S rRNA Cardinium* | 54 ºC / 1:30min | 450 | PCR |
| ArsF^29^  ArsR2^29^ | GGGTTGTAAAGTACTTTCAGTCGT  GTAGCCCTRCTCGTAAGGGCC | *16S rRNA Arsenophonus* | 60 ºC / 1:30min | 800 | PCR |
| 16STF1^30^  TKSSsp^24^ | GGTCTTCGGATTGTAAAGGTCTG TAGCCGTGGCTTTCTGGTAA | *16S rRNA*  *Spiroplasma* | 56 ^o^C / 30sec | 94 | qPCR |
| GmmtubqF^31^  GmmtubqR^31^ | CCATTCCCACGTCTTCACTT  GACCATGACGTGGATCACAG | host *β-tubulin* | 56 ^o^C / 30sec | 151 | qPCR |
| FqdnaA^32^  RqdnaADoud (This study) | TGAAAAAAACAAACAAATTGTTATTACTTC  TTAAGAGCAGTTTCAAAATCAGG | dnaA | 56 ^o^C / 30sec | 138 | qPCR |
| 63F^24^  16STR1_Hasel^30^ | GCCTAATACATGCAAGTCGAAC  GGTGTGTACAAGACCCGAGAA | *16S rRNA*  *Spiroplasma* | 67 ºC/ 1:30min | 1334 | PCR, SR |
| 16F2_Bi^33^  23R1_Bi^33^ | GGTGCATGGTTGTCGTCAG  TTCGCTCGCCGCTACTAAG | region 16S-23S  *Spiroplasma* | 67 ºC / 1min | 1024 | PCR, SR |
| 23F1_Bi^33^  5R_Bi^33^ | GAATGGGGAAACCCGGTGAG  TCGGGATGGGAACGGGTG | region 23S-5S  *Spiroplasma* | 67 ºC / 3min | 2914 | PCR, SR |
| 23F2_BiDoud (This study) | ATAGACCCGAAACCAGGTGA | region 23S-5S  *Spiroplasma* | - | - | SR |
| 23F3_Bi^33^ | CCGTGAGGACTGCTGGACTG | region 23S-5S  *Spiroplasma* | - | - | SR |
| 23F3_Heres^34^ | CCTAAGGCAAGCGAGATAAC | region 23S-5S  *Spiroplasma* | - | - | SR |
| 23R2_Bi^33^ | GGATCACTAAGCCCAGCTTT | region 23S-5S  *Spiroplasma* | - | - | SR |
| fru-f^35^  fru-r^35^ | GTCATAATTGCAATTGCTGG  CAATGATTAAAGCGGAGGT | *fruR*  *Spiroplasma* | 56 ºC / 30sec | 398 | PCR, SR |
| SRdnaAF1^36^  SRdnaAR1^36^ | GGAGAYTCTGGAYTAGGAAA  CCYTCTAWYTTTCTRACATCA | *dnaA*  *Spiroplasma* | 50 ºC / 30sec | 515 | PCR, SR |
| FtsZF2^30^  FtsZR3^30^ | TGAACAAGTCGCGTCAATAAA  CCACCAGTAACATTAATAATAGCATCA | *ftsZ*  *Spiroplasma* | 57 ºC / 1min | 774 | PCR, SR |
| ParEF2^30^  ParER2^30^ | GGAAAATTTGGTGGTGATGG  TGGCATTAATCATTACATTAATTTCT | *pare*  *Spiroplasma* | 57 ºC / 1min | 1126 | PCR, SR |
| RpoBF1^30^  RpoBR2^30^ | ATGGATCAAACAAATCCATTAGCAGA  GCATGTAATTTATCATCAACCATGTGTG | *rpoB*  *Spiroplasma* | 60 ºC / 1:30min | 1703 | PCR, SR |
| RpoBR4^30^ | CTTTGTTTCCATGGCGTCCAGCC | *rpoB*  *Spiroplasma* | - | - | SR |


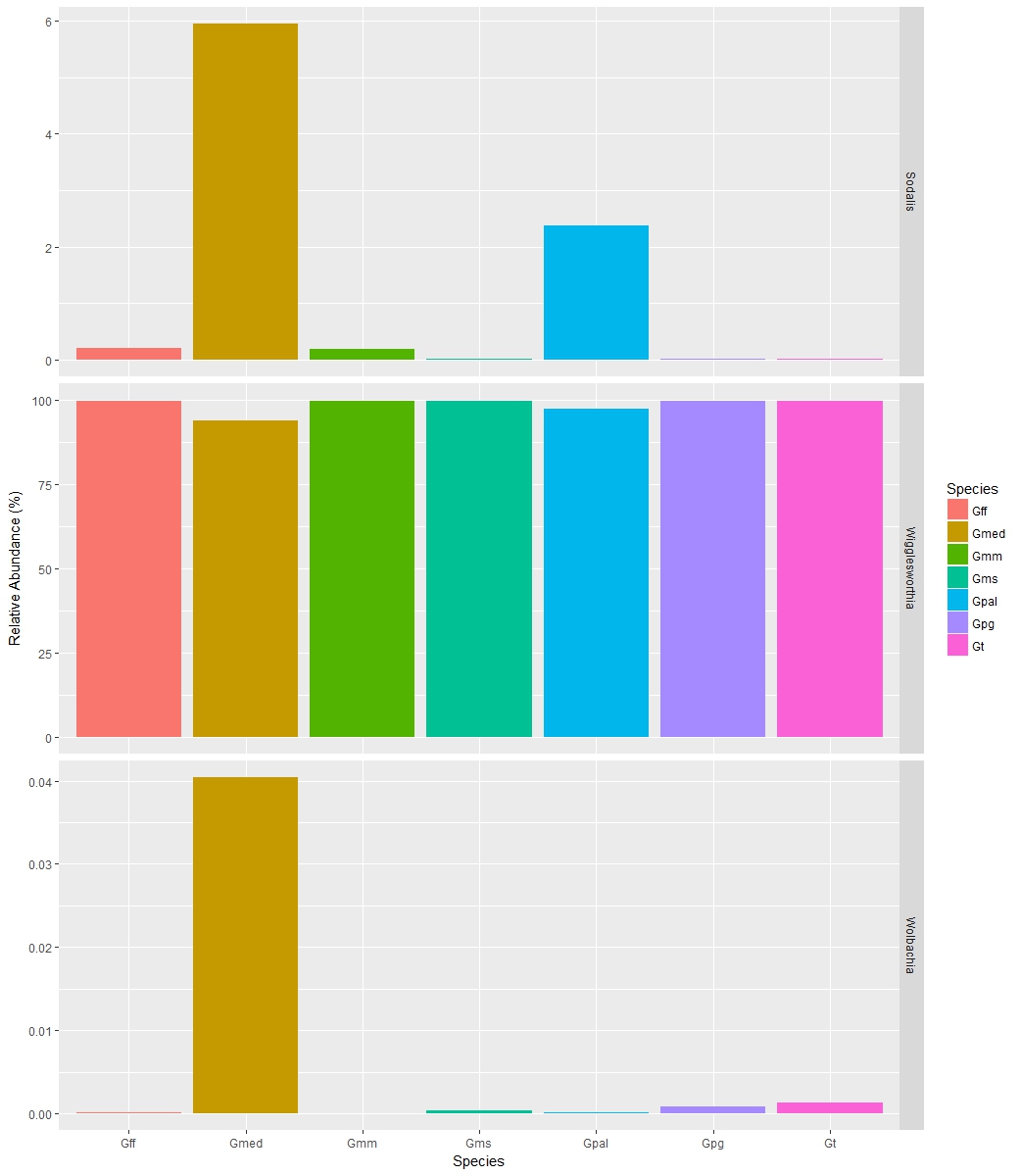


**Supplementary Figure 1** Relative abundance of *Wigglesworthia*, *Sodalis*, and Wolbachia, in whole wild tsetse flies. (Gff: *G. fuscipes fuscipes* (n=76); Gmed: *G. medicorum* (n=8); Gmm: *Glossina* *morsitans* *morsitans* (n=6), Gms: *G. morsitans submorsitans* (n=8); Gpal: *G. pallidipes* (n=42); Gpg: *G. p. gambiensis*  (n=8); Gt: *G. tachinoides*) (n=8).


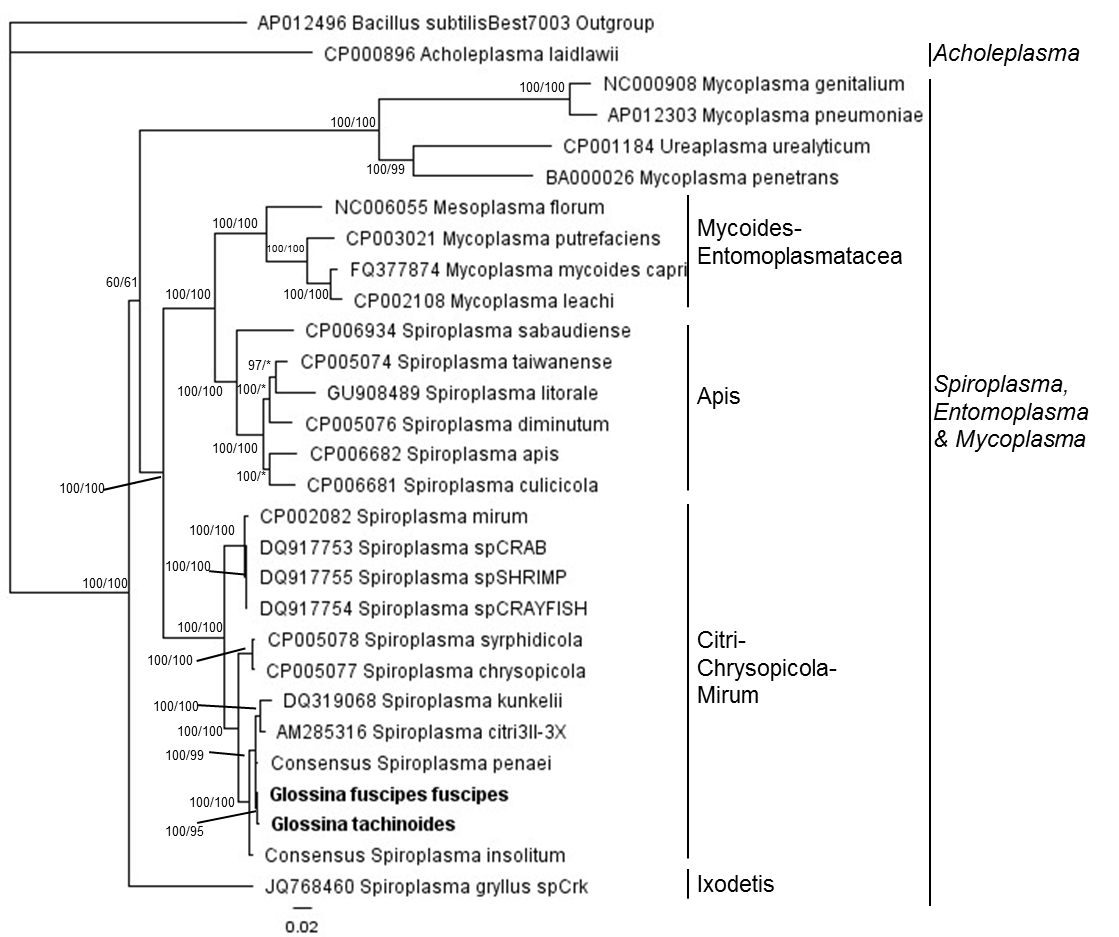


**Supplementary Figure 2** Bayesian inference phylogeny based on the **16S-23S-5S rRNA** sequence: The topology resulting from the Maximum Likelihood (ML) method was similar. Bayesian posterior probabilities and ML bootstrap values based on 1000 replicates are given at each node (only values >50% are indicated), respectively. Asterisks indicate support values lower than 50%. The *Spiroplasma* strains present in *Gff* and *Gt* are indicated in bold letters. For each *Spiroplasma* species the GenBank accession number is given to the left of the name.


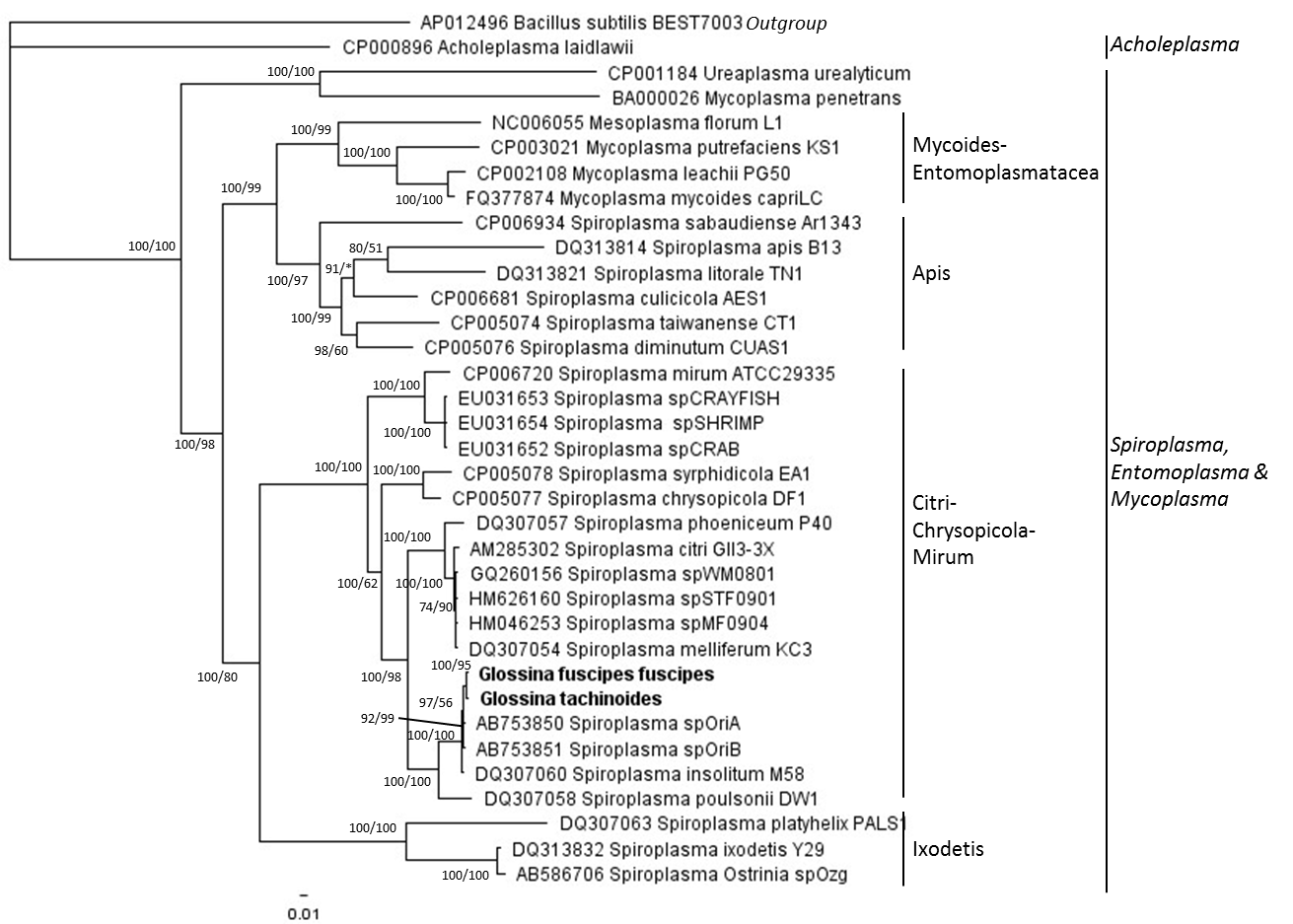


**Supplementary Figure 3** Bayesian inference phylogeny based on the ***rpoB*** sequence: The topology resulting from the Maximum Likelihood (ML) method was similar. Bayesian posterior probabilities and ML bootstrap values based on 1000 replicates are given at each node (only values >50% are indicated), respectively. Asterisks indicate support values lower than 50%. The *Spiroplasma* strains present in *Gff* and *Gt* are indicated in bold letters. For each *Spiroplasma* species the GenBank accession number is given to the left of the name.


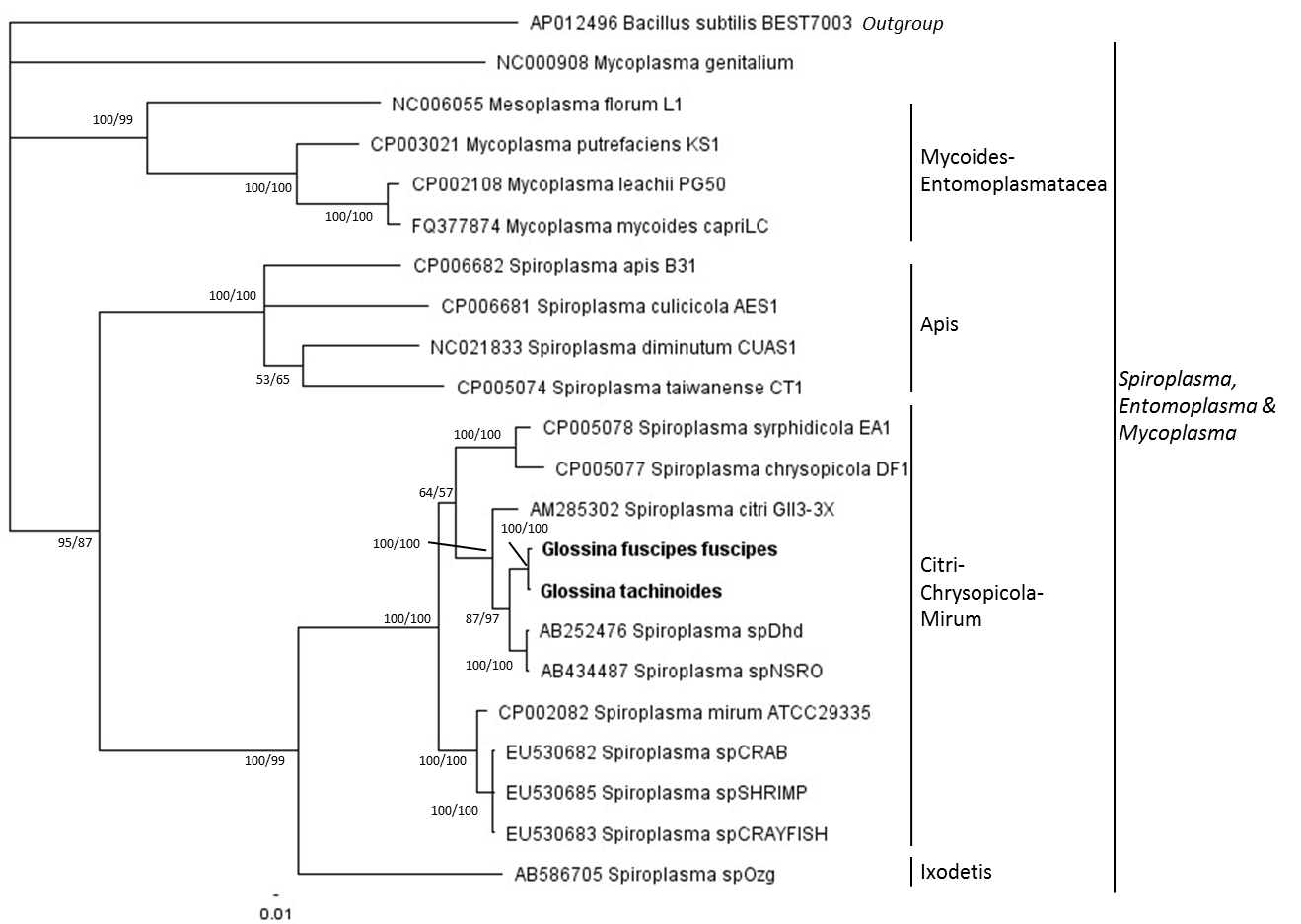


**Supplementary Figure 4** Bayesian inference phylogeny based on the ***dnaA*** sequence: The topology resulting from the Maximum Likelihood (ML) method was similar. Bayesian posterior probabilities and ML bootstrap values based on 1000 replicates are given at each node (only values >50% are indicated), respectively. Asterisks indicate support values lower than 50%. The *Spiroplasma* strains present in *Gff* and *Gt* are indicated in bold letters. For each *Spiroplasma* species the GenBank accession number is given to the left of the name.


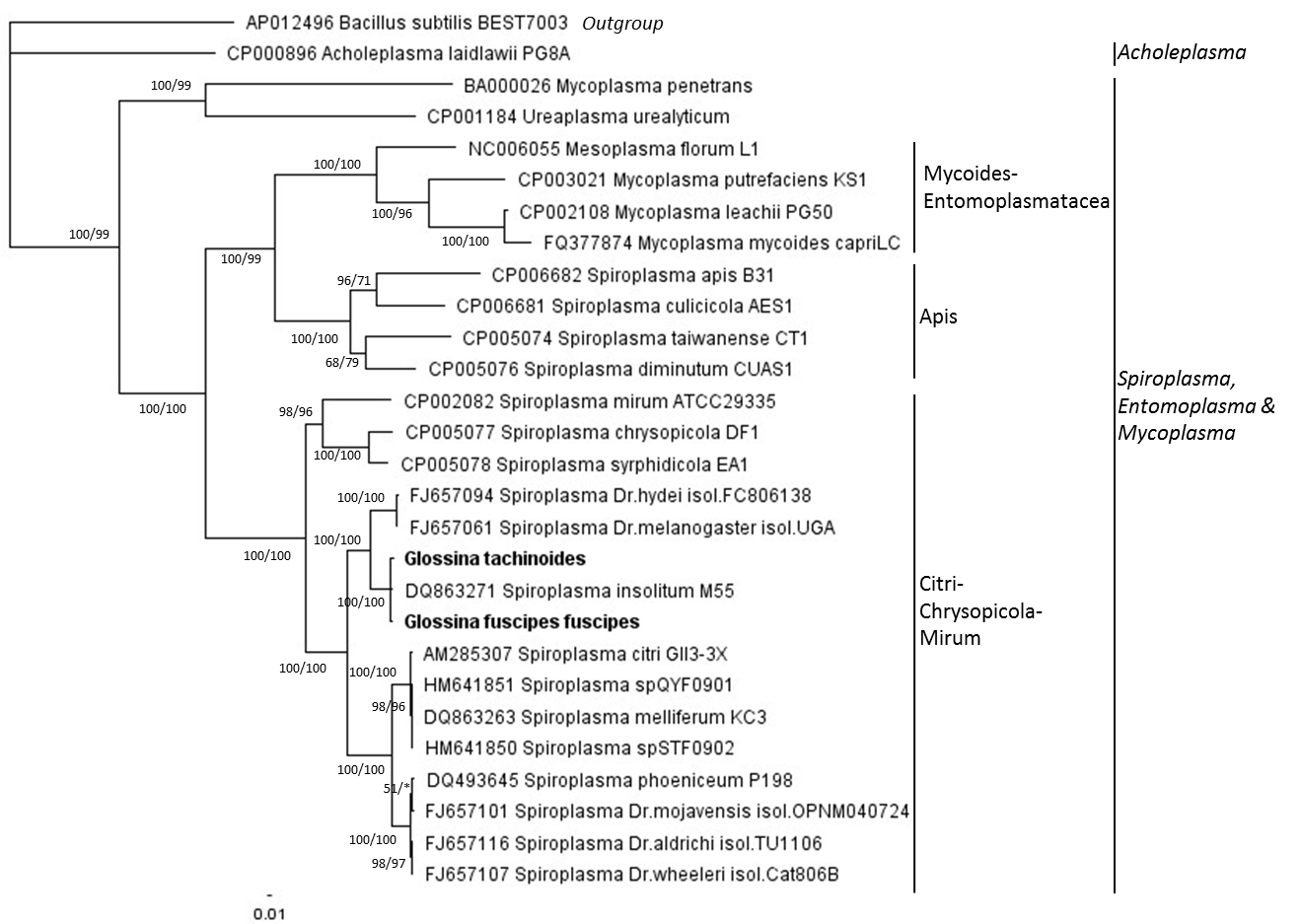


**Supplementary Figure 5** Bayesian inference phylogeny based on the ***parE*** sequence: The topology resulting from the Maximum Likelihood (ML) method was similar. Bayesian posterior probabilities and ML bootstrap values based on 1000 replicates are given at each node (only values >50% are indicated), respectively. Asterisks indicate support values lower than 50%. The *Spiroplasma* strains present in *Gff* and *Gt* are indicated in bold letters. For each *Spiroplasma* species the GenBank accession number is given to the left of the name.


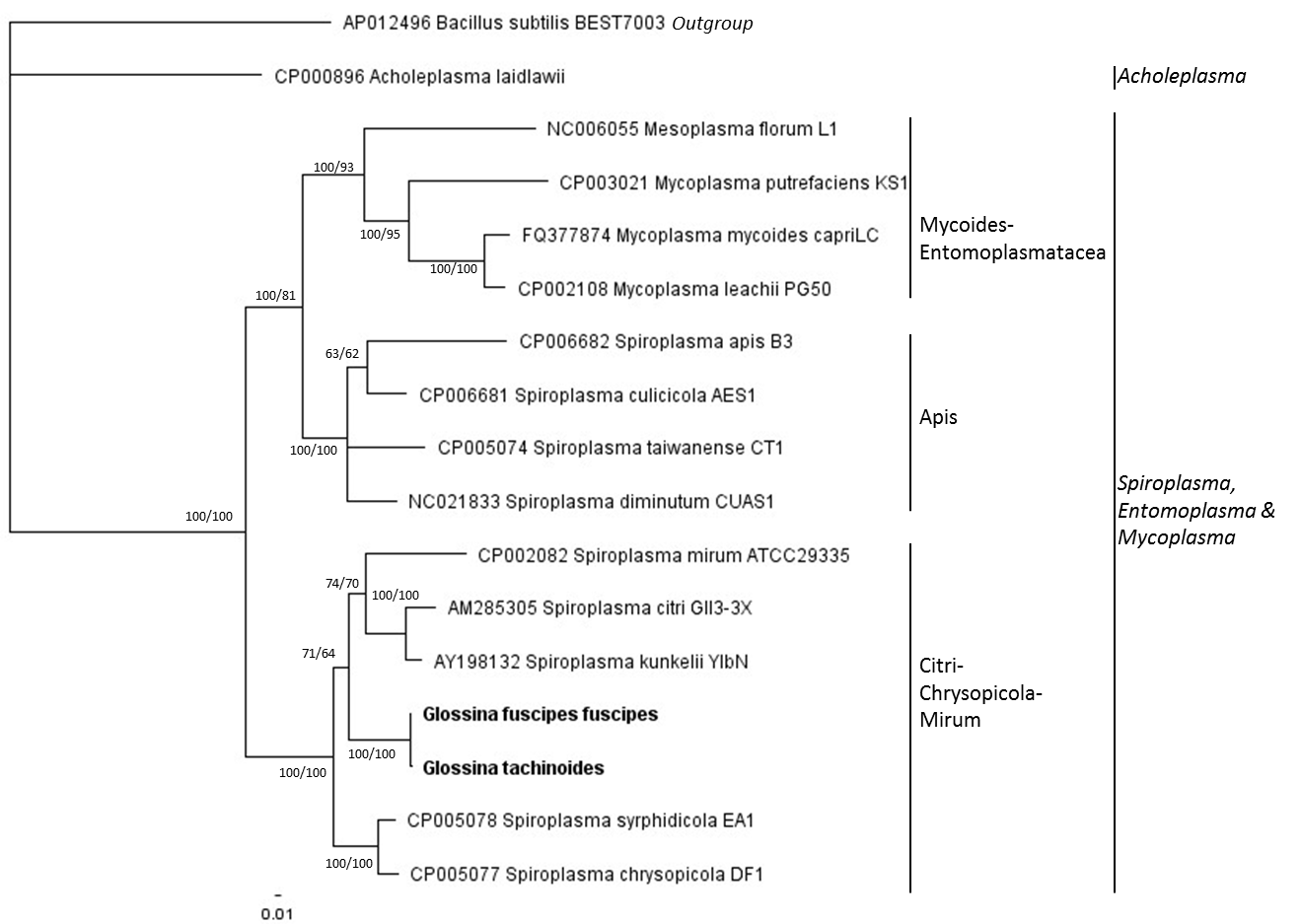


**Supplementary Figure 6** Bayesian inference phylogeny based on the ***ftsZ*** sequence: The topology resulting from the Maximum Likelihood (ML) method was similar. Bayesian posterior probabilities and ML bootstrap values based on 1000 replicates are given at each node (only values >50% are indicated), respectively. Asterisks indicate support values lower than 50%. The *Spiroplasma* strains present in *Gff* and *Gt* are indicated in bold letters. For each *Spiroplasma* species the GenBank accession number is given to the left of the name.


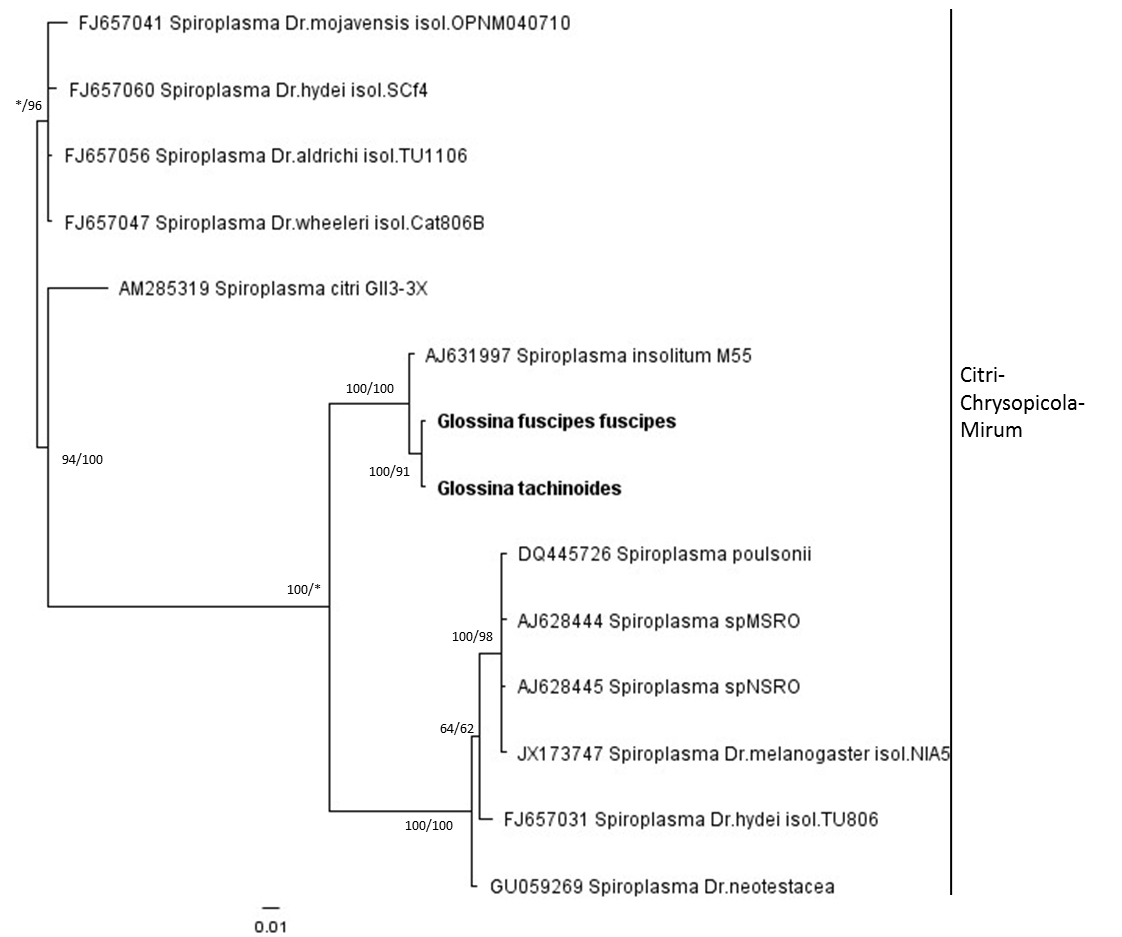


**Supplementary Figure 7** Bayesian inference phylogeny based on the ***fruR*** sequence: The topology resulting from the Maximum Likelihood (ML) method was similar. Bayesian posterior probabilities and ML bootstrap values based on 1000 replicates are given at each node (only values >50% are indicated), respectively. Asterisks indicate support values lower than 50%. The *Spiroplasma* strains present in *Gff* and *Gt* are indicated in bold letters. For each *Spiroplasma* species the GenBank accession number is given to the left of the name.


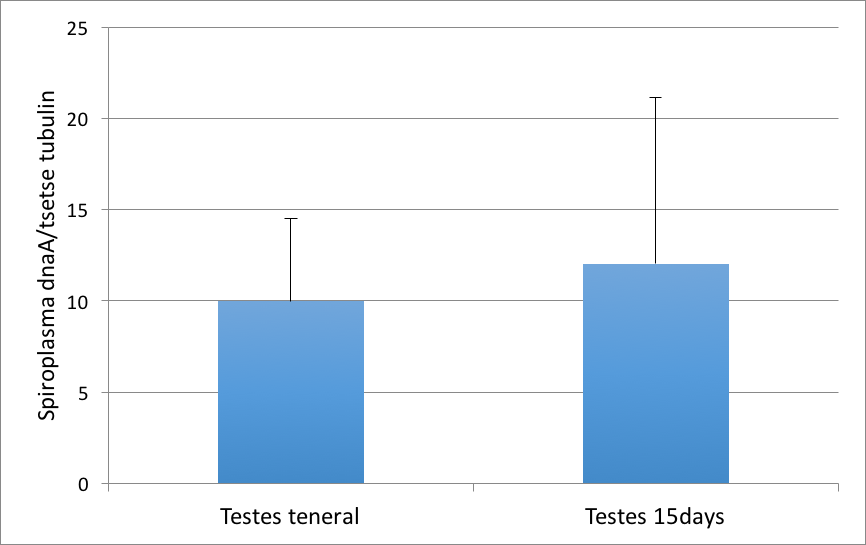


**Supplementary Figure 8** Quantification of *Spiroplasma* titer in terms of the symbiont *dnaA* gene copies normalized by the tsetse β-tubulin gene. *Gff* testes from teneral and 15-day old flies (n=3, each sample is a pool of five).


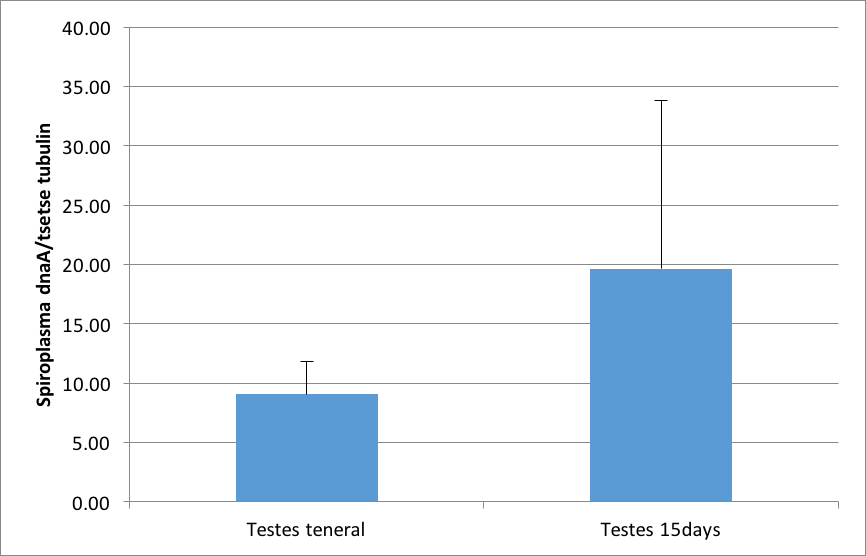


**Supplementary Figure 9** Quantification of *Spiroplasma* titer in terms of the symbiont *dnaA* gene copies normalized by the tsetse β-tubulin gene. *Gff* ovaries from teneral and 15-day old flies (n=3, each sample is a pool of five).
